# Blood metabolomic and transcriptomic signatures stratify patient subgroups in multiple sclerosis according to disease severity

**DOI:** 10.1016/j.isci.2024.109225

**Published:** 2024-02-15

**Authors:** Alexandra E. Oppong, Leda Coelewij, Georgia Robertson, Lucia Martin-Gutierrez, Kirsty E. Waddington, Pierre Dönnes, Petra Nytrova, Rachel Farrell, Inés Pineda-Torra, Elizabeth C. Jury

**Affiliations:** 1 Division of Medicine, Department of Inflammation, University College London, London WC1E 6JF, UK; 2 Scicross AB, Skövde, Sweden; 3 Department of Neurology and Centre of Clinical, Neuroscience, First Faculty of Medicine, General University Hospital and First Faculty of Medicine, Charles University in Prague, 500 03 Prague, Czech Republic; 4 Department of Neuroinflammation, University College London and Institute of Neurology and National Hospital of Neurology and Neurosurgery, London WC1N 3BG, UK

**Keywords:** Classification Description, Molecular network, Metabolomics, Transcriptomics, Machine learning

## Abstract

There are no blood-based biomarkers distinguishing patients with relapsing-remitting (RRMS) from secondary progressive multiple sclerosis (SPMS) although evidence supports metabolomic changes according to MS disease severity. Here machine learning analysis of serum metabolomic data stratified patients with RRMS from SPMS with high accuracy and a putative score was developed that stratified MS patient subsets. The top differentially expressed metabolites between SPMS versus patients with RRMS included lipids and fatty acids, metabolites enriched in pathways related to cellular respiration, notably, elevated lactate and glutamine (gluconeogenesis-related) and acetoacetate and bOHbutyrate (ketone bodies), and reduced alanine and pyruvate (glycolysis-related). Serum metabolomic changes were recapitulated in the whole blood transcriptome, whereby differentially expressed genes were also enriched in cellular respiration pathways in patients with SPMS. The final gene-metabolite interaction network demonstrated a potential metabolic shift from glycolysis toward increased gluconeogenesis and ketogenesis in SPMS, indicating metabolic stress which may trigger stress response pathways and subsequent neurodegeneration.

## Highlights

• Metabolite signature accurately discriminated RRMS from patients with SPMS
• The signature out-performed retrospective clinical markers of disease severity
• Patients with SPMS characterized by disrupted lipid metabolism and cellular respiration
• Gene-metabolite network shows increased gluconeogenesis/ketogenesis in SPMS

## Graphical abstract

**Figure:**
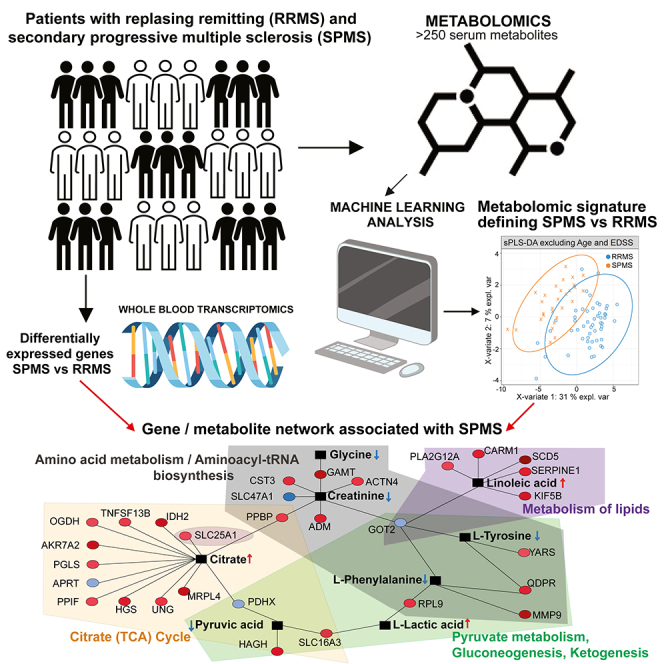

## Introduction

Multiple sclerosis (MS) is an autoimmune disease with both inflammatory and neurodegenerative components, affecting ∼2.5 million people worldwide. Approximately 85% of patients with MS are diagnosed with the relapsing remitting form of the disease (RRMS), characterized by reversible or near reversible periods of neurological impairment (relapses). RRMS is characterized by systemic auto-inflammation resulting in neuronal demyelination and oligodendrocyte and neuro-axonal injury that may or may not be associated with increased disability.^1^^,^^2^^,^^3^ Most patients with RRMS eventually move to a more progressive disease phase associated with brain and spinal cord atrophy, where disability increases and relapses become less likely (secondary-progressive MS, SPMS).^1^^,^^4^ The pathogenic mechanisms driving the accrual of disability in progressive disease are less well known and include neuro-axonal, astrocyte, and oligodendrocyte damage leading to neurodegeneration, likely mediated by compartmentalized chronic inflammation within the central nervous system (CNS).^1^^,^^2^^,^^3^^,^^5^ It is now accepted that progression leading to significant, irreversible disability is due to both “relapse-associated worsening” and “progression independent of relapse activity” and is seen in almost all people with MS over time.^6^^,^^7^

Although the pathogenesis of these two clinical phases of MS is different, the transition from RRMS to SPMS is a diagnostic challenge, there are no validated imaging or biofluid biomarkers that can distinguish between these two phases of MS, and as a result, SPMS diagnosis is made retrospectively following irreversible disability accrual. This has implications for appropriate treatment strategies, as there are many treatments approved for treating patients with RRMS but very few disease modifying therapies for SPMS have been approved. A growing number of studies show that people with MS are characterized by altered blood metabolomic and transcriptomic profiles.^8^^,^^9^^,^^10^ Blood metabolites assessed using various nuclear magnetic resonance (NMR) and mass spectroscopy platforms can discriminate between people with MS compared to age and sex matched healthy donors^11^^,^^12^ and other neurodegenerative diseases,^13^ as well as between patients with SPMS from RRMS.^14^^,^^15^ Analysis of the transcriptomic profiles in the blood and brain of patients with RRMS versus SPMS also supports altered molecular processes between these patient groups.^9^^,^^16^^,^^17^^,^^18^^,^^19^

Thus, a better understanding of the molecular landscape in the blood of patients with RRMS versus SPMS could facilitate a more personalized approach to treatment from the time of diagnosis, thereby delaying/reducing the accumulation of progressive disability. Biomarkers identifying/predicting disease severity could also help to assess the efficacy of new drugs and improve clinical trial design. This study combined analysis of serum metabolomics and whole blood transcriptomics in patients with RRMS compared to SPMS to build an integrated network describing these different phases of MS to improve understanding of the potential mechanisms driving disease progression.

## Results

### Serum metabolites can stratify between multiple sclerosis patient subgroups and healthy and disease controls

Sera from patients with relapsing-remitting MS (RRMS, n = 52), secondary progressive MS (SPMS, n = 29), healthy donors (HCs, n = 80), and patients with neuromyelitis optica (disease controls - DCs, n = 30, an autoantibody-mediated disease that shares some symptoms and may be misdiagnosed as MS) were analyzed using an NMR serum metabolomic platform (Table S1 for participant demographics; Table S2 for list of metabolites). Metabolomic data were compared between groups using seven machine learning (ML) models (Figure S1 for the study plan).^20^ All ML models discriminated between groups with high accuracy, with the ensemble model bagged logistic regression (LR) showing the best performance discriminating between patients with HCs and RRMS (accuracy: 0.871, area under the curve - receiver operator characteristic analysis (AUC ROC): 0.920) and the boosted LR model showing the best performance discriminating between patients with HCs and SPMS (accuracy: 0.945, AUC ROC: 0.910) (Tables 1 and S3). Similar stratification was observed between MS patient groups and DCs (Tables 1 and S4).

**Table 1:** Metabolites distinguish between patients with RRMS and SPMS from healthy and disease controls with high accuracy

Comparison	Model	Accuracy	Sensitivity	Specificity	AUC ROC
HC vs. DC	**Bagged LR**	**0.918**	**0.867**	**0.938**	**0.958**
	Boosted LR	0.855	0.568	0.963	0.920
HC vs. RRMS	**Bagged LR**	**0.871**	**0.888**	**0.846**	**0.920**
	Boosted LR	0.849	0.846	0.850	0.891
HC vs. SPMS	Bagged LR	0.927	0.925	0.931	0.930
	**Boosted LR**	**0.945**	**0.897**	**0.963**	**0.959**
DC vs. RRMS	Bagged LR	0.817	0.865	0.733	0.846
	**Boosted LR**	**0.829**	**0.865**	**0.767**	**0.910**
DC vs. SPMS	**Bagged LR**	**0.949**	**0.931**	**0.967**	**0.993**
	Boosted LR	0.932	0.931	0.933	0.984

Performance statistics for the top two performing models comparing RRMS, SPMS cohorts with healthy and disease controls. Ensemble methods gave best performance: bagged and boosted logistic regression (LR). Sensitivity (true positive rate); specificity (true negative rate). Statistics are rounded to 3 decimal places. HC, healthy controls; DC, disease control (neuromyelitis optica). See Table S3 and S4 for list of top differentiating features.

**Figure 1:**
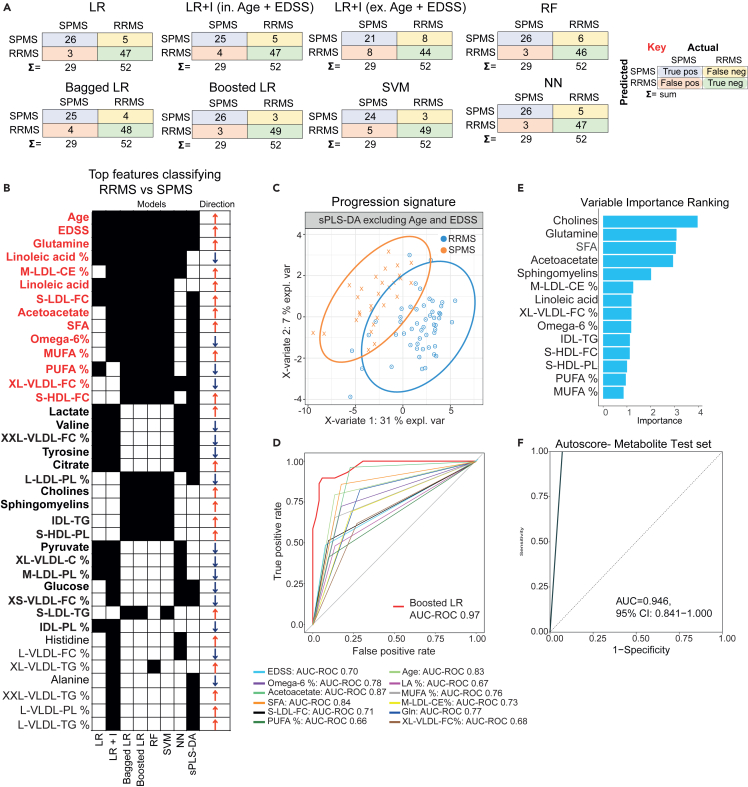
Serum metabolomics can stratify patients with SPMS vs. RRMS Related to Table 2, Figure S2 and Tables S5–S8. NMR serum metabolomics analysis in patients with SPMS (n = 29) and RRMS (n = 52). Metabolomic signatures associated with SPMS vs. RRMS determined using machine learning. (A) Confusion matrices showing number of correct (blue squares) and incorrect (green squares) classifications for each model. The sum (Σ) of each row and column is given. The models were: logistic regression (LR) with/without interactions (I) including/excluding age and EDSS (expanded disability status score), bagged and boosted LR, support vector machine (SVM), random forest (RF), neural network (NN) and sparse partial least square discriminant analysis (sPLS-DA). See Figures S2A and S2B. (B) Comparison of metabolites selected by each machine learning model (black squares). Metabolites selected by > 6 models highlighted in red bold; metabolites selected by ≥ 3 models in black bold. (C) sPLS-DA plot (sparse partial least squares-discriminant analysis) to validate metabolomic signature in (B) in metabolites identified by ≥ 3 models. See Figure S2D for features in components 1 and 2. (D) Area under the curve-Receiver operator characteristic (AUC-ROC) of top 14 metabolites and/or clinical features identified by ≥ 6 models and the best performing machine learning model, boosted LR. See Table S2 for metabolite abbreviations. (E and F) Metabolite features in (B) analyzed using the Youden Index to identify features that best stratify patients with SPMS vs. RRMS. Features with Youden J statistic >0.5 were analyzed using the Autoscore model. (E) Importance ranking of top variables (See Tables S7 and S8). (F) AUC-ROC plot showing performance of autoscore test set.

ML analysis of metabolomic data was also able to stratify between patients with SPMS vs. RRMS (Table 2). Overall, LR and random forest (RF) models performed best when predicting SPMS cases, correctly identifying 26 out of 29 (89.7%) patients (Figure 1A). While boosted LR performed better for RRMS cases, with excellent specificity (0.961) and an accuracy of 93.67% (Table 2). A significant separation between SPMS and RRMS patient groups was also observed using a sparse partial least squares-discriminant analysis (sPLS-DA) model (Figures S2A and S2B).

**Table 2:** Comparison of predictive model performance of SPMS vs. RRMS

Model	Accuracy	Sensitivity	Specificity	AUC ROC
LR	0.901	0.897	0.904	0.939
LR + I (in. Age and EDSS)	0.889	0.862	0.904	0.936
LR + I (ex. Age and EDSS)	0.803	0.724	0.846	0.844
Bagged LR	0.901	0.862	0.923	0.920
Boosted LR	0.926	0.897	0.942	0.965
RF	0.889	0.897	0.885	0.939
SVM	0.901	0.828	0.942	0.930
NN	0.901	0.897	0.904	0.939

Refer to Figure 1. Performance statistics for 8 predictive models based on serum metabolites and clinical features for the SPMS vs. RRMS comparison. The models used were Lasso LR with and without interactions (I) including and excluding age and EDSS, the ensemble methods: bagged and boosted LR, SVM, RF, and NN. The sensitivity represents the true positive rate (SPMS) in contrast to specificity, which is the true negative rate (RRMS). Statistics are rounded to 3 decimal places. AUC ROC indicates area under the receiver operating characteristic curve; LR, logistic regression; RF, random forest; and SVM, support vector machine.

To derive a signature that could discriminate SPMS from patients with RRMS, features were compared across all analyses (Figure 1B). Thirty-six measures including metabolites, lipids, and clinical demographic information were featured in three models or more. The top features included glutamine (Gln) and the clinical features age and EDSS, which appeared in all seven models as well as in the sPLS-DA models (Figures 1B, S2A, and S2B). Linoleic acid (LA), cholesterol esters in medium low-density lipoprotein (M-LDL-CE), free cholesterol in small low-density lipoprotein (S-LDL-FC), acetoacetate, saturated fatty acid (SFA), omega-6 fatty acid (%),mono- and poly unsaturated fatty acids (MUFA/PUFA %) and free cholesterol in extra-large very low-density lipoprotein (XL-VLDL-FC %) were identified by six ML models and/or sPLS-DA (Figures 1B and S2C). Furthermore, glycolysis-associated metabolites (lactate, glucose, pyruvate, and citrate), amino acids (valine, tyrosine, histidine, and alanine), and sphingomyelin were featured in three ML models or more (Figure 1B). The full list of metabolites identified by a minimum of one ML model is listed in Table S5.

As expected, age and EDSS, known to be associated with severity and progression, were identified by all the models (Figure 1B). The influence of age and EDSS was further examined using the LR with interactions (LR + I) model, which was performed with and without age and EDSS features (Figures 1A; Table 2; Table S6). Although excluding age and EDSS decreased model accuracy (88.9%–80.3%) and AUC ROC (0.936–0.844), metabolites alone were still capable of classifying patients with SPMS and RRMS with good performance (Table 2). The ability of metabolomic features identified by three or more ML models (n = 29, excluding age and EDSS) to classify SPMS from patients with RRMS, was confirmed by sPLS-DA (Figure 1C; Figure S2D for loadings on components 1 and 2). AUC ROC curve analysis showed that the best performing ML model (Boosted LR, Table 2) outperformed the individual top ranked metabolites (identified by six models or more from Figure 1B) and patient features age and EDSS when discriminating between patients with SPMS vs. RRMS (Figure 1D).

To develop a robust method to stratify RRMS from patients with SPMS based on serum metabolomics, the optimum cut-off value was calculated for each serum metabolite identified in >4 ML models (Youden Index,^21^; Table S7). Features with a Youden Index >0.5 were selected and used to build a points-based system for stratifying patient groups (Autoscore^22^). The autoscore model (training dataset - 70% of patients) identified the optimum cut-off values for the top five features (cholines, glutamine, saturated fatty acids, acetoacetate, and sphingomyelins) (Figure 1E for the importance ranking of the features; Table S8). The performance of the final model was confirmed in the test set (30% of patients) which stratified RRMS from SPMS with an AUC ROC of 0.9464 (Figure 1F). This analysis demonstrated that serum metabolites could be used to develop a reliable method to stratify patients, which could potentially out-perform clinical markers of severity such as EDSS and age.

### Patients with secondary progressive multiple sclerosis have altered glucose, lipid, and amino acid metabolism compared to patients with relapsing-remitting multiple sclerosis

**Figure 2:**
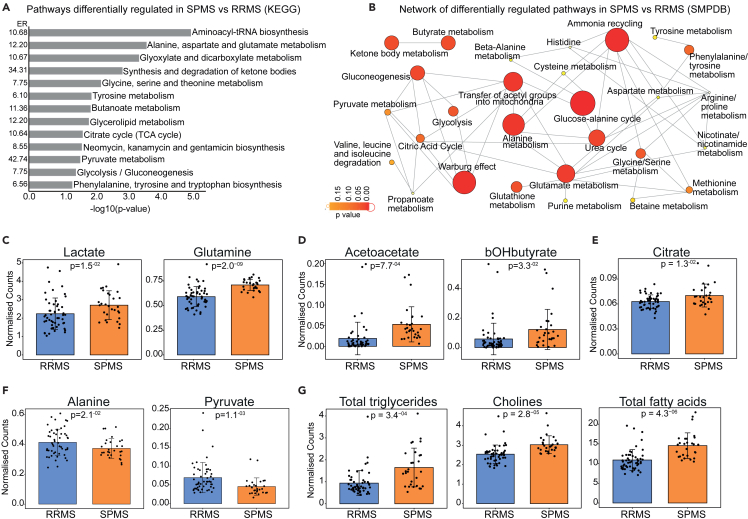
Metabolite set enrichment analysis (MSEA) on SPMS vs. RRMS metabolomic signature Related to Table S5. Metabolites identified as associated with SPMS by > 1 model (Table S5) were analyzed by MSEA. (A) Enrichment analysis using KEGG database. Pathways with a significant enrichment p < 0.05 are shown (–log10(p value)). Enrichment ratio (ER) is indicated along y axis. (B) Network of metabolic pathways derived from SMPDB database. Nodes are sized using the -log10(p value) and colored on a red to yellow gradient. The larger and redder the node, the greater the significance of the p value. (C–G) Bar charts showing the relative expression of metabolites (normalised values) in serum from patients with RRMS n = 52 (blue) vs. SPMS n = 29 (orange) (C) lactate and glutamine, representative metabolites of gluconeogenesis pathway; (D) acetoacetate and bOHbutyrate (ketogenesis pathway) (E) citrate (TCA); (F) alanine and pyruvate (glycolysis-related) and (G) total triglycerides, cholines and total fatty acids associated with lipid metabolism. T-tests were performed to identify statistically significant differences between patients with RRMS (blue) and patients with SPMS (red). p value, mean and +/− SD shown.

To assess whether the metabolites identified by ML models indicated differences in metabolic processes between patients with RRMS versus SPMS, a metabolite set enrichment analysis was performed (Figures 2A and 2B). Metabolic pathways related to protein synthesis, amino acid and lipid metabolism, and cellular respiration were enriched in patients with SPMS compared to RRMS, including “Synthesis and degradation of ketone bodies”/“Ketone body metabolism” (KEGG: p = 1.27E-03, SMPDB: p = 1.62E-02), “Glycerolipid metabolism” (KEGG: p = 1.41E-02), “Gluconeogenesis” (KEGG: p = 3.57E-02, SMPDB: p = 1.51E-02) and other cellular-respiration associated pathways including Glycolysis (KEGG: p = 3.57E-02, SMPDB: p = 5.57E-02), “Pyruvate metabolism” (KEGG: p = 2.61E-02, SMPDB: p = 1.70E-01) and “Citric Acid Cycle” (KEGG: p = 2.18E-02, SMPDB: p = 8.65E-02) (Figures 2A and 2B). Network analysis showed that these pathways were related (Figure 2B) and support a potential change in cellular respiration in SPMS compared with patients with RRMS characterized by increased gluconeogenesis and ketogenesis, as shown previously.^23^^,^^24^^,^^25^ This observation was supported by comparing serum metabolite concentrations: gluconeogenesis-related metabolites (lactate and glutamine), ketone bodies (acetoacetate and bOHbutyrate), and citrate (citric acid cycle) (Figures 2C–2E) were significantly elevated in patients with SPMS compared to RRMS, while glycolysis-related metabolites (alanine and pyruvate) were reduced (Figure 2F). Lipid (e.g., triglycerides, choline) and fatty acid metabolites were also dysregulated in SPMS compared with patients with RRMS (Figure 2G). Overall, these data show a complex change in the serum metabolic signature between patients with SPMS and RRMS.

### Differential metabolite expression is mirrored in the whole blood transcriptome in patients with secondary progressive multiple sclerosis compared to relapsing-remitting multiple sclerosis

**Figure 3:**
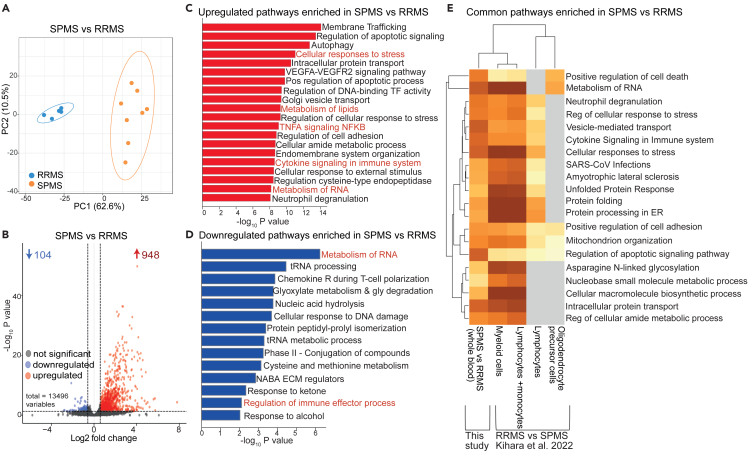
Metabolism of RNA, cellular responses to stress, immune effector response, cellular respiration, metabolism of lipids and tRNA processing pathways are dysregulated between patients with SPMS and RRMS Related to Figure S3 and Tables S9 and S10. RNA-sequencing was performed on n = 8 patients with SPMS and n = 5 patients with RRMS followed by differential gene expression and pathway enrichment analysis. Refer to Figures S3A–S3F. (A) Principal component analysis on all 1052 DEGs clustering patients with SPMS (orange) from patients with RRMS (blue). (B) Volcano plot showing differentially expressed genes (DEGs) Log2 fold change (>1.5 or < -1.5) and FDR-adjusted p value<0.05. Colored points represent significantly up- (red) and down- (blue) regulated genes in SPMS compared to RRMS. (C and D) Pathway enrichment analysis of DEGs analyzed by Metascape to identify regulated pathways. (C) Bar chart of top 20 significantly enriched pathways upregulated in patients with SPMS (Gene Ontology (GO), Reactome, Hallmark, Wikipathways). Pathways are ranked by p value. (D) Bar chart of top 14 significantly enriched pathways downregulated in patients with SPMS (Gene Ontology (GO), Reactome, Hallmark, Wikipathways). Pathways are ranked by p value. (E) Validation of gene expression pathways using an independent dataset. Gene lists were taken from.^17^ This was performed on multiple cell types including, myeloid cells, lymphocytes, whole blood (myeloid and lymphocytes combined) and oligodendrocyte precursor cells. For consistency only genes with a log2FC of 0.585 and FDR<0.05 was considered.

To evaluate whether changes in the serum metabolome were reflected more widely, whole blood RNA-sequencing was performed on a subset of patients. Differential expression analysis comparing MS patient groups identified 1052 differentially expressed genes (DEGs), which were able to cluster SPMS from patients with RRMS (Figure 3A). Of these, 948 genes were upregulated and 104 downregulated (Figure 3B). The top 20 up and down regulated DEGs are listed in Table S9.

Pathway enrichment analysis of up- and down-regulated genes in SPMS vs. RRMS also identified pathways associated with cell metabolism, including “metabolism of RNA,” “cellular response to stress,” “metabolism of lipids,” and “cellular respiration” (Figures 3C and 3D; Figures S3A–S3E; Table S10 for DEG lists). In total, 215 DEGs were associated with the above-mentioned metabolic pathways (Table S11). This includes the genes: nuclear respiratory factor 1 (NRF1), C-C chemokine receptor type 5 (CCR5), Glutamic- oxaloacetic transaminase 2 (GOT2), mitoregulin (LINC00116/MTLN) and O-sialoglycoprotein endopeptidase (OSGEP), which form part of the list of the top 20 up and downregulated genes (Table S9). Immune activation pathways including “TNFA signaling” and “cytokine signaling in the immune system,” and “regulation of immune effector processes” were also differentially enriched in patients with SPMS compared with RRMS (Figures 3D and 3E; Figures S3A and S3F). Inflammatory mechanisms are known to be different between the two disease phases, as demonstrated by altered serum cytokine expression in RRMS compared to progressive MS and TNF-α-induced oligodendrocyte cell death, supporting demyelination in progressive disease.^26^^,^^27^ Interestingly, network analysis of the “cellular respiration pathway” revealed an association with “Amyotrophic lateral sclerosis” (Figure S3G),^28^ supporting a role for dysregulated cell metabolism in the neurodegenerative processes in SPMS.

Finally, many of the metabolic and immune-associated pathways enriched in patients with SPMS compared to RRMS were also identified when compared to an independent gene expression dataset comparing whole blood, lymphocytes, myeloid cells, and oligodendrocyte precursor cells from SPMS compared with patients with RRMS (Figure 3E).^17^

Together, these results indicated a systemic alteration in metabolic pathways in patients with SPMS compared to patients with RRMS focused on RNA biosynthesis, lipid metabolism, and cellular respiration pathways (Figures 2A, 3C, 3D, and S3).

### Gene-metabolite interaction network proposes a potential mechanism behind the metabolic switch in multiple sclerosis severity

The relationship between metabolites and DEGs in matched samples was further explored using correlation analysis. Multiple significant correlations (p < 0.01) were identified between metabolites and DEGs in the cellular respiration, regulation of immune effector response pathways, metabolism of lipids, and metabolism of RNA (Figures S4A–S4D). Most of these correlations were positive, and the large number of correlations suggest a coordination between metabolites and genes associated with cellular respiration and metabolism from a transcriptomic to metabolomic level.

**Figure 4:**
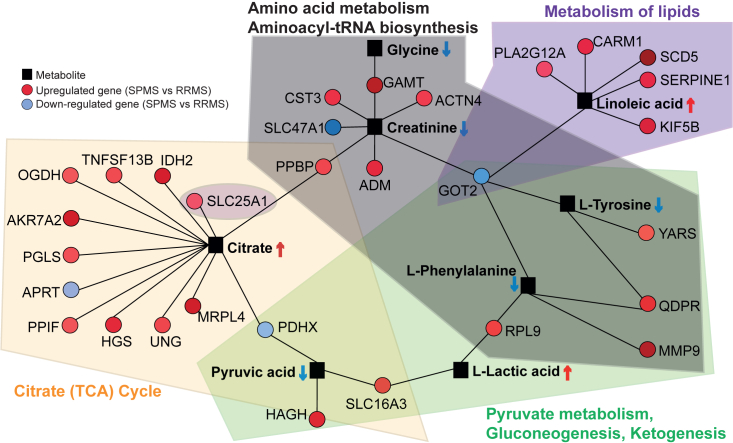
Gene-metabolite interaction network Related to Table S12. Gene-metabolite interaction network was constructed using the network analysis tool in MetaboAnalyst. This was performed by combining all 1052 DEGs and any metabolite that was identified by the machine learning models. The gene-metabolite interaction network illustrates the crosstalk between 27 upregulated (red circles) and 4 downregulated genes (blue circles) with 7 metabolites from the metabolomic signature (black squares). Genes are also colored based on the strength of their fold-change. Whilst phenylalanine was not identified as part of the metabolomic signature, it has been found to be lowered in MS, in previous publications. These genes and metabolites are associated with the metabolic pathways: aminoacyl-tRNA biosynthesis, glycolysis/gluconeogenesis, pyruvate metabolism and the TCA cycle.

To further integrate the metabolomic and transcriptomic data, network analysis was performed on all 1052 DEGs and the SPMS metabolomic signature. Within the final network, 26 upregulated and four downregulated genes were found to interact with eight metabolites (creatinine, citrate, pyruvate, lactate, phenylalanine, tyrosine, glycine, and linoleic acid) from the metabolomic signature (Figure 4; Table S12 for the function of genes within the gene-metabolite interaction network). Although phenylalanine was not identified by this analysis, it has been found to be deficient in patients with MS previously.^11^ While lipids could not be included within the network, the upregulated genes SCD5 (Stearoyl-CoA Desaturase 5), PLA2G12A (Phospholipase A2 Group XIIA), CARM1 (Coactivator Associated Arginine Methyltransferase 1) and SLC25A1 (Soluble Carrier Family 25 Member 1) were within the metabolism of lipid pathway. The downregulated gene GOT2 (log2FC: −0.617) is associated with multiple metabolic pathways, including amino acid metabolism, aminoacyl tRNA biosynthesis, metabolism of lipids, pyruvate metabolism, gluconeogenesis, and ketogenesis, and has been previously associated with MS.^29^ Taken together, this gene-metabolite interaction network demonstrates the crosstalk between key genes and metabolites, which could highlight molecular processes associated with MS severity. The results suggest a potential metabolic switch from glycolysis toward increased gluconeogenesis and ketogenesis, supported by the fatty acid β-oxidation and conversion of ketogenic and gluconeogenic amino acids, phenylalanine, and tyrosine, in patients with SPMS compared to those with RRMS (Figure S5).

## Discussion

This study used a combination of analysis methods to show that patients with RRMS could be stratified from patients with SPMS based on serum metabolomic profiles, which included changes in lipid, amino acid, ketone body, and other metabolites, confirming previous reports.^30^^,^^31^^,^^32^ Furthermore, a putative score was developed that could be used for stratification between MS patient subsets, which if validated, supports the use of metabolite signatures for diagnosis, treatment decisions, and clinical trial design. More detailed analysis identified that the SPMS metabolomic profile was enriched for metabolites within the gluconeogenesis and ketogenesis pathways but reduced in glycolysis-related metabolites, potentially reflecting cellular respiration changes in patients with SPMS compared to RRMS. This was corroborated using whole blood transcriptomic analysis, which identified genes enriched in the “cellular response to stress,” “metabolism of lipids,” and “cellular respiration” pathways in patients with SPMS compared to RRMS. Finally, multiple correlations between SPMS-associated metabolites and genes were identified, and an interaction network between key differentially expressed genes and metabolites highlighted glycolysis/gluconeogenesis, pyruvate metabolism, the TCA cycle, and aminoacyl-tRNA biosynthesis to be associated with MS severity.

MS can be difficult to differentiate from other neurological diseases, diagnosis is largely based on clinical symptoms and MRI results^33^ and there are no validated blood-derived biomarkers for MS diagnosis or for the differentiation of RRMS from progressive disease^34^. Identifying proxy measures of the neuronal manifestations of MS beyond MRI imaging (which provides a retrospective indication of disease severity) and cerebral spinal fluid (CSF) sampling via lumber puncture, represents a clinical need.^35^ Several potential biomarkers assessing neuronal damage and inflammation and measurable in serum have been proposed in MS.^36^^,^^37^ Serum levels of neurofilament light chains (NfL, released into the CSF following axonal damage and neuronal death) are highly correlated with CSF NfL measurements in matched samples, and longitudinal serum NfL measurements can be used to predict MS disease activity/progression, including new and enlarging T2 lesions and brain volume loss in individual patients.^34^ Other biomarkers present in both in serum and CSF that have been proposed for assessing MS disease prognosis include, glial fibrillary acidic protein (a marker of neuronal damage), and inflammation markers osteopontin, CXCL13, and CD163.^36^ In this study, supervised analysis of serum metabolomic data using ML models identified a metabolite signature that could stratify RRMS from patients with SPMS; healthy individuals from patients with patients with RRMS and SPMS, and neuromyelitis optica (similar to MS in both clinical and radiological presentation) from patients with RRMS and SPMS, all with high accuracy. This suggests that metabolomic signatures could be used to aid MS diagnosis and define specific disease endotypes that could aid patient stratification and personalized medicine. This finding is supported by previous studies using supervised ML approaches for analysis of metabolomic datasets.^38^^,^^39^^,^^40^^,^^41^ However, despite the many previous studies no unique metabolite signature has been defined, likely due to the use of different metabolomic platforms and difficultly in developing clinical metabolomic assays.^30^^,^^31^

The advantage of the work presented here is that an established, clinical grade, and standardized metabolomic platform was employed. This platform has been validated with a high correlation against both clinical metabolite standards and against standard detection methodology, namely clinical chemistry autoanalyzer measures.^42^ This platform has also been used to assess metabolomic signatures in several large biobanks including the UK biobank^42^ and FINRISK biobanks^43^ for biomarker research and translation. Our study uses this reproducible and cost effective metabolomic platform to compare patients with SPMS vs. RRMS to better define the heterogeneity of MS. Furthermore, using this platform could support future validation of the SPMS-associated metabolite signature in existing MS patient cohorts, providing an opportunity to identify and develop new diagnostic and prognostic serum biomarkers for SPMS as well as provide insight into systemic molecular therapeutic targets.

Our study also suggests that phenotyping disease stage in patients with MS could be improved by incorporating additional measures of key pathological processes identified in this study, such as changes in lipid metabolism and increased ketogenesis observed in SPMS. For example, ketone bodies including bOHbutyrate and acetoacetate were both increased in SPMS. Interestingly, acetoacetate alone (AUC ROC 0.87) outperformed EDSS (AUC ROC 0.70) and age (AUC ROC 0.83) when discriminating between patients with SPMS and RRMS. Similar studies report a systemic elevation of acetoacetate and bOHbutyrate in the CSF and blood from patients with MS compared to controls.^40^^,^^44^^,^^45^ Ketone bodies can cross the blood-brain barrier so may be produced in the brain and then eliminated into the blood where they can be detected.^46^ The strong association of ketone bodies with SPMS could suggest that monitoring serum levels may have the potential to pin-point if a patient is likely to progress before irreversible disability accrual. However, it remains to be validated whether ketone bodies, (e.g., acetoacetate) are systemic biomarkers that could add to the list of serum biomarkers for precision phenotyping in MS. Work is ongoing to relate serum and CSF measures of these metabolites.

The use of multi-omics (metabolomic and transcriptomic) can also shed light on the heterogeneous pathology associated with SPMS. Previous studies have used similar approaches, for example analysis of spatial transcriptomics and proteomics in fresh frozen brain tissue from patients with progressive MS identified molecular pathways associated with neurodegeneration that were not targeted by current therapeutic strategies.^47^ In this study, Kaufmann and colleagues identified that myelination is downregulated in oligodendrocyte precursor cells, a finding that may correlate with elevated serum sphingomyelin within our metabolomic signature.^47^ Integration of multi-omics data using unsupervised techniques (e.g., Multi-Omics Factor Analysis^48^ and Data Integration Analysis for Biomarker Discovery using Latent components^49^) on a range of omic datasets (metabolomics, bulk, single cell, and spatial transcriptomics, proteomics, and so forth) could also help to identify putative drivers of heterogeneity in SPMS.^50^^,^^51^ Applying this type of analysis across multiple tissues/cell types (blood, CSF, and brain tissues) to map pathogenesis and identify similarities in disease mechanisms/markers may well help to characterize and validate reliable biomarkers of severity to help clinicians with diagnosis (which is currently retrospective after a certain period of irreversible disability accrual has occurred) and implementing interventions.^50^ Furthermore, identifying blood-based biomarkers would help to avoid more invasive techniques for diagnosis and monitoring.

In addition, this type of granular interrogation of the molecular landscape in patients with MS could help to identify disease endotypes, patients who maybe similar clinically but may respond to treatments in different ways, for a more personalized medicine approach.^34^ For example, in depth analysis of blood-based immune phenotype and serum metabolomics has identified blood-based signatures associated with the future development of anti-drug antibodies to interferon-beta in patients with RRMS.^52^^,^^53^ This type of information could be used to guide treatment decisions as well as help patient selection to future clinical trials to improve outcomes.^34^ Finally, this multi-omic approach has the potential to identify new drug targets that can be validated in experimental models of disease as well as in other human datasets.^47^^,^^54^

It should be noted that omic data has some limitations. For example metabolomic data can be influenced by factors such as age, diet,^55^ and hormonal status^56^ and transcriptomic analysis does not always translate to changes in protein expression or activity due to RNA silencing and post-transcriptonal modifications.^57^ The analysis platform selected can also influence the reproducibility of the omic data obtained. For metablomics, NMR is more cost effective (meaning more samples can be analyzed), more reproducible, and requires minimal sample preparation compared to mass spectroscopy methods, although the number of molecules measured by NMR is more limited.^58^ For transcriptomic data, batch to batch variation can make some omic data difficult to replicate without appropriate controls. Furthermore, as mentioned above, the use of single omics only provides a partial insight into biological processes and does not reflect the molecular complexities of biological systems.^51^ Multi-omic analysis provides a better understanding of the biological system, however, omic integration requires matched samples, which are not always available, meaning that improved cohort and experimental planning is needed to fully leverage the advantages that omic analysis can provide.^51^^,^^54^

Serum cholesterol (LDL and VLDL cholesterol subsets, M-LDL-CE %, S-LDL-FC, and XL-VLDL-FC%) and fatty acids (MUFA/PUFA %, Omega-6%, SFA, and LA) were important features differentiating directly between patients with SPMS and RRMS. There is growing evidence that altered levels of cholesterol and cholesterol derivatives in blood and CSF could be both implicated in the pathogenesis of MS, and be used clinically as biomarkers of disease activity, disease progression, and response to treatment.^59^^,^^60^ Elevated total and LDL cholesterol are associated with increased disease progression assessed by T2 lesion development (a measure of brain tissue damage) and the EDSS score.^61^^,^^62^ VLDL-FC is also reported to be highly correlated with the EDSS disability score in patients with MS.^63^ A recent metabolomic analysis using a PLS-DA model also identified lipoproteins as among the top features discriminating between patients with RRMS and SPMS, including HDL/LDL and VLDL which were all lower in SPMS.^41^ In experimental models, defects in cholesterol efflux to HDL can inhibit the remyelination processes in the brain and contribute to disability and progression.^64^ Elevated serum HDL is associated with lower rates of disease progression in MS.^59^ We found that HDL subsets were largely elevated in SPMS compared to RRMS, although this does not necessarily reflect the function of HDL, which can be inflammatory.^65^ Overall, our findings largely correspond to previous reports, differences are likely due to the metabolomic platform used, whereby a more detailed and complex breakdown of lipoprotein subsets is examined in the current study.^42^

Fatty acid metabolism is also known to be disrupted in patients with MS in general,^66^ with differences also reported between patients with RRMS and SPMS.^31^ A recent study showed that both plasma omega-3 and omega-6 fatty acids were associated with measures of disability in patients with MS, including MRI measures of brain volume and serum biomarkers NfL and glial fibrillary acidic protein.^67^ Furthermore, patients with RRMS and progressive MS could be distinguished based on their distinct serum fatty acid derivative profiles,^68^ supporting our findings that fatty acids are important features for discriminating between MS patient subgroups. Arachidonic acid is an omega-6 fatty acid that can be metabolized into pro-inflammatory prostaglandins and leukotrienes which are known to be elevated in the brain of patients with MS.^69^ Notably, patients with progressive MS had increased levels of arachidonic acid derivatives^67^ and arachidonic acid derivatives correlated positively with EDSS scores, while omega-3 fatty acid derivatives had more variable associations with markers of disability and progression.^68^ Interestingly, LA is a PUFA that is metabolized to omega-3 fatty acids and arachidonic acid, and both LA concentration and the ratio of LA (LA %) among total fatty acids were among the top features differentiating between patients with SPMS and RRMS. However, while in this study LA concentration was increased suggesting the conversion of LA to omega-3 fatty acids may be impaired in SPMS, another study showed that gamma-LA was reduced in patients with progressive MS.^15^ Overall, these results support a role for blood metabolites within the cholesterol and fatty acid metabolism pathways as reliable biomarkers for disease subtype stratification.

The dyslipidemia identified in our metabolomic analysis may indicate that patients with SPMS have increased cardiovascular disease (CVD) risk compared to patients with RRMS. This observation agrees with a study showing that patients with more severe or progressive disease are older and have more CVD events compared to patients with RRMS.^70^ Hypertension and heart disease are also associated with brain atrophy development in patients with MS.^71^ Thus, lowering CVD-risk may also reduce the risk of increased brain atrophy and progression in patients with SPMS. Patients with SPMS may benefit from lifestyle changes that reduce CVD-risk including diet^72^ or alternative strategies for lowering serum lipid levels and CVD-risk such as statins. Interestingly, high dose simvastatin, a CNS-penetrant statin, attenuates brain atrophy and disease progression in patients with SPMS^73^ and a phase-III clinical trial testing the efficacy of simvastatin in SPMS is in progress (MS-STAT2; NCT03387670, http://www.isrctn.com/ISRCTN82598726). Of note, the mechanism of action of statins in SPMS may go beyond lipid lowering with wider effects on immune activation and signaling reported.^74^

Pathway and network analysis of metabolomic and transcriptomic data also identified potential molecular mechanisms associated with the pathogenic processes in patients with RRMS compared with SPMS. The metabolomic signatures suggested that ketone body and glutamate metabolism, and gluconeogenesis were enriched in patients with SPMS compared to RRMS, supporting a potential change in cellular respiration toward increased gluconeogenesis and ketogenesis and defects in glycolysis in SPMS compared with RRMS. This was supported by the whole blood transcriptomic analysis. DEGs were enriched in pathways associated with the metabolism of RNA, cellular respiration, and lipid metabolism as well as various immune-associated pathways. Several studies have examined the blood and/or brain tissue transcriptomic profiles in patients with RRMS and SPMS either compared with HCs or each other.^16^^,^^17^^,^^75^^,^^76^ One study compared PBMC microarray data from untreated patients with RRMS and SPMS ^75^ and similar to our findings, immune pathways and lipid and arachidonic acid metabolism were dysregulated between the two patient subgroups. Another microarray analysis of whole blood between patients with RRMS and primary progressive MS also identified differences in metabolic pathways including a down regulation of the oxidative phosphorylation pathway and an upregulation of RNA metabolism-associated pathways in progressive MS.^76^ Interestingly, when we compared DEGs from whole blood (this study) with those obtained from SPMS brain tissue (oligodendrocyte precursor cells, monocytic cells, and lymphocytes),^17^ several overlapping pathways were highlighted relevant to cellular responses to stress, metabolism of RNA, cellular amide metabolic process, and regulation of apoptotic signaling.^17^ This supports the systemic nature of MS disease pathology, whereby there is overlap between gene expression and metabolic pathways between whole blood and cells from brain tissue from patients with SPMS.

In summary, this study shows that serum metabolites could be attractive candidate biomarkers to identify patients with MS that have transitioned to a more severe/progressive disease. Currently, there are no validated imaging or biofluid biomarkers that can distinguish between patients with RRMS and SPMS and this remains a diagnostic challenge. Metabolites have both diagnostic and prognostic potential to improve SPMS diagnosis, which is currently made retrospectively following irreversible disability accrual. Finally, the results presented here could indicate a systemic alteration in metabolic pathways in patients with SPMS compared to patients with RRMS focused on RNA biosynthesis, lipid metabolism, and cellular respiration pathways that could provide insight into the pathogenic mechanisms associated with these two phases of the disease.

### Limitations of the study

This study had some limitations including an unbalanced sample size between patients with RRMS compared to controls and patients with SPMS for the metabolomic analysis and a small sample size for the transcriptomic analysis. Despite this, the data reported here are supported by previous reports investigating differences in the molecular landscape between patient subtypes in MS 30,31. This this study builds on the body of evidence supporting a metabolitc shift occurring as neurodegeneration progresses. Differences in demographic information were seen between the groups (where available), this was accounted for in the ML models, in particular age, and EDSS would be expected to be different between patients with SPMS compared to RRMS. Notably, our analysis shows that despite age and EDSS being important features in the stratification, metabolites alone are also important features. Another limitation is that patients with SPMS had been treated with disease modifying therapies before their transition from RRMS to SPMS, whereas the RRMS cohort were recruited before first treatment. However, previous studies have shown that metabolites can discriminate between these patient subsets, independent of treatment regimens.^14^ The multiple studies investigating metabolite signatures in MS all support the use of metabolites as a potentially valuable tool for future diagnostic/prognostic tests. This study attempted to convert the metabolite signature associated with SPMS into a putative score that could be used for stratification. This score would need to be validated in further cohorts, however this work supports the use of metabolite signatures using an established methodology that can be scaled for routine use.

## STAR★Methods

### Key resources table

**Table:** 

REAGENT or RESOURCE	SOURCE	IDENTIFIER
**Biological samples**		

Peripheral blood (serum and whole blood) from patients with multiple sclerosis	Department of Neurology and Center of Clinical, Neuroscience, First Faculty of Medicine, General University Hospital, Prague, Czech Republic and National Hospital of Neurology and Neurosurgery, London, WC1N 3BG, United Kingdom	N/A
Peripheral blood (serum) from patients with neuromyelitis optica	Department of Neurology and Center of Clinical, Neuroscience, First Faculty of Medicine, General University Hospital, Prague, Czech Republic and	N/A
Peripheral blood (serum) from healthy donors	University College London Hospital and University College London	N/A

**Deposited data**		

Serum metabolomics data	This study	Mendeley: https://data.mendeley.com/datasets/t26rn8hth8/1
Whole blood RNA-sequencing	This study	ArrayExpress Accession number: E-MTAB-13378 Release date: 2024-09-19
Single nucleus RNA-seq on SPMS vs. RRMS patients	Kihara et al.^17^	https://doi.org/10.3389/fncel.2022.918041

**Software and algorithms**		

R package mixOmics	Rohart et al.^77^	https://doi.org/10.1371/journal.pcbi.1005752
RStudio 4.2.0	R Core Team (2021)87	https://www.R-project.org
MetaboAnalyst 5.0	Chong and Xia.^21^	https://doi.org/10.1007/978-1-0716-0239-3_17
AutoScore	Xie et al.^22^	https://doi.org/10.2196/21798
DESeq2	Love et al.^78^	http://www.bioconductor.org/packages/release/bioc/html/DESeq2.html
Metascape	Zhou et al.^79^	https://doi.org/10.1038/s41467-019-09234-6
ClustVis	Metsalu and Vilo.^80^	https://doi.org/10.1093/nar/gkv468
Cytoscape	Shannon et al.^81^	https://doi.org/10.1101/gr.1239303
DisGeNET	Piñero et al.^82^	https://academic.oup.com/nar/advance-article/doi/10.1093/nar/gkz1021/5611674
glmnet R package	Friedman et al.^83^	Friedman et al.^83^
LogicFS	Ruczinski et al.^84^	https://doi.org/10.1198/1061860032238
Caret R package	Kuhn.^85^	https://doi.org/10.18637/jss.v028.i05
CaTools	Tuszynski.^86^	https://cran.r-project.org/package=caTools
R package ggplot2	Wickham.^87^	https://doi.org/10.1111/j.1541-0420.2011.01616.x
Circlize	Gu et al.^88^	https://doi.org/10.1093/bioinformatics/btu393
Cocor	Diedenhofen and Musch.^89^	https://doi.org/10.1371/journal.pone.0131499
EnhancedVolcano	Blighe et al.^90^	https://github.com/kevinblighe/EnhancedVolcano.
RColorBrewer	Neuwirth.^91^	https://CRAN.R-project.org/package=RColorBrewer
Ggpubr	Kassambara.^92^	https://CRAN.R-project.org/package=ggpubr
Pheatmap	Kolde.^93^	https://CRAN.R-project.org/package=pheatmap

**Additional resources**		

EU Clinical Trials Register	Anti-Biopharmaceutical Immunization: Prediction and analysis of clinical relevance to minimize the risk of immunization in multiple sclerosis patients on interferon-beta treatment	https://www.clinicaltrialsregister.eu/ctr-search/trial/2012-005450-30/SE

### Resource availability

#### Lead contact

Further information and requests for resources should be directed to and will be fulfilled by the lead contact Professor Elizabeth Jury (e.jury@ucl.ac.uk).

#### Materials availability

This study did not generate new unique reagents.

#### Data and code availability

Metabolomic data have been deposited at Mendeley and are publicly available as of the date of publication. The DOI is listed in the key resources table.

Whole blood RNA-seq data have been deposited at GEO and are publicly available as of the date of publication. Accession numbers are listed in the key resources table.

Any additional information required to reanalyze the data reported in this paper is available from the lead contact upon request.

This paper does not report original code.

### Experimental model and study participant details

Peripheral blood was collected from patients with relapsing-remitting MS (RRMS, n = 52) (age, median 34 years, range 19–59; sex, female n = 37, male n = 15; ethnicity, n = 52 white) and secondary progressive MS (SPMS, n = 29) (age, median 54 years, range 35–78; sex, female n = 18, male n = 11; ethnicity, n = 26 white, n = 2 Black/Caribbean, n = 1 mixed/other), diagnosed according to the revised McDonald criteria.^94^ Patients with RRMS were disease modifying treatment naive. Clinical information including age, sex, ethnicity, smoking status, body mass index (BMI) and expanded disability status score (EDSS) was recorded. Smoking status was categorised as never smoked, quit or current smoker. As controls peripheral blood from healthy donors (HCs, n = 80) (age, median 36 years, range 19–76; sex, female n = 56, male n = 24; ethnicity, n = 54 white, n = 7 Black/Caribbean, n = 11 Asian/Indian, n = 1 mixed/other, n = 7 unknown) and patients with neuromyelitis optica (disease controls - DCs, n = 30, an autoantibody-mediated disease that shares some symptoms and may be misdiagnosed as MS) (age, median 48 years, range 23–66; sex, female n = 25, male n = 5), ethnicity (n = 30 unknown) was also collected.

Demographic data (age, sex, ethnicity) was included in machine learning models when possible. Multiple sclerosis is a disease that has a female predominance (female:male ratio 3:1).

Ethical approvals for this work were obtained from the University College London Hospitals National Health Service Trust research ethics committee (Reference numbers 18/SC/0323 – RELOAD-MS study; 15-LO-2065, 16/YH/0306, and 15/SW/0109- ABIRISK study) and the Medical Ethics Committee of the General University Hospital in Prague (125/12 and Evropský grant 1.LF UK-CAGEKID). All participants provided informed written consent in accordance with the Declaration of Helsinki. Patient and control characteristics are detailed in Table S1 (total cohort and RNA-sequencing sub-cohort). The study design is summarised in Figure S1.

### Method details

#### Serum metabolomics

Measures of 250 serum biomarkers were acquired with a well-established nuclear magnetic resonance (NMR)-spectroscopy platform (Nightingale Health, using 350 μL serum platform) (Table S2). These included both absolute concentrations, percentages, and ratios of lipoprotein composition. Serum lipids measured included apolipoproteins (Apo) and (very) low density ((V)LDL), intermediate density (IDL) and high density (HDL) lipoprotein particles of different sizes ranging from chylomicrons and extremely large (XXL), very large (XL), large (L), medium (M), small (S), and very small (XS).

This metabolomic platform provides clinical grade analysis that is fast, cost-effective, and reproducible. In our analysis, we have used serum vials which have not been exposed to freeze/thaw cycles (FTCs), despite good evidence of serum metabolome stability on NMR spectroscopy analysis after 1–5 FTCs.^95^ The success rate of biomarker quantification was >99% across the cohort and metabolite concentrations fell within the distributions commonly observed in general population cohorts.

Metabolomic data is available on Mendeley: https://data.mendeley.com/datasets/t26rn8hth8/1.

#### RNA preparation and sequencing

Whole blood RNA was isolated from five patients with RRMS and eight patients with SPMS using Qiagen PAXgene Blood RNA extraction kit. Sample concentration and purity was determined using a NanoDropTM 1000 Spectrophotometer. Sequencing, quality control analysis, genome alignment, and quantification was performed by UCL Genomics from 50 ng of isolated RNA/sample (London, UK). Ilumina technology was used to produce high-throughput sequencing data. This data was processed by Picard,^96^ aligned to the GRCH38 homo sapiens reference genome by STAR,^97^ and processed and filtered by fastp.^98^

#### Differential gene expression analysis

Differential gene expression between RRMS and SPMS patients was determined by DESeq2-1.36.0 analysis using Wald hypothesis testing and parametric fit typing and using the Benjamin-Hochberg method for multiple test correction.^78^ No genes were removed using the DESeq2 filtering process which made the p value adjustment more stringent. Using the entirety of the gene set, principal component analysis (PCA) was used to reduce the dimensionality of the dataset and to visualise the level of clustering and separation between RRMS and SPMS patients. Lastly, using a reduced number of genes, after applying a threshold of ≥25 counts, volcano plots were generated to visualise the differential expression analysis results. As well as to identify the number of genes which passed a threshold of both FDR<0.05 and FC±1.5 (log2FC: 0.585).

Pathway enrichment analysis was performed using the web-based portal Metascape on the differentially expressed gene (DEG) list using the inclusion threshold. The following databases were used: Gene ontology (GO); KEGG; Reactome; BioCarta; WikiPathways; Canonical and Hallmark databases.^79^ To visualise differences in expression levels between SPMS and RRMS patients, heatmaps depicting normalised gene counts for gene sets of interests from top biological pathways were created using ClustVis [https://biit.cs.ut.ee/clustvis/].^80^ Both rows and columns were clustered using Euclidean distance and Ward linkage and trees were ordered using the tightest cluster first.

Data available at ArrayExpress accession no. E-MTAB-13378.

#### Predictive models

To derive a serum metabolomic signature that could discriminate MS subtypes (RRMS and SPMS) from HCs and DCs and to stratify RRMS from SPMS, metabolites were compared across seven Machine learning (ML) models: logistic regression (LR), LR with interactions (LR + I); bagged and boosted LR; random forest (RF), support vector machine (SVM) and neural network (NN); sparse Partial Least Squares Discriminant Analysis (sPLS-DA) with or without interactions (see below for details of models).

##### Imputation

Missing values were imputed using k-nearest neighbors (kNN), a non-parametric classification algorithm assigning an unknown observations class based on the class of a number (k) of similar observations within the feature space. The default value of *k* = 5 was used.

##### Homology reduction

Many of the metabolites measured are biologically interdependent, and therefore highly correlated. To reduce homology, if two features had a correlation coefficient >0.95 then the feature with the greatest mean absolute correlation with the remaining features was removed.

##### Data scaling

Metabolites were centered on the mean and scaled to the standard deviation.

##### Predictors

The independent variables included in the models were the homology reduced datasets for the following comparisons: HC vs. DC (113 metabolites), HC vs. RRMS (113 metabolites), HC vs. SPMS (119 metabolites), DC vs. RRMS (105 metabolites), DC vs. SPMS (112 metabolites) and SPMS vs. RRMS (111 metabolites), as well as the cohort information (age, sex, BMI, smoking status, ethnicity, and baseline EDSS).

##### Model performance

10-fold cross-validation was used to evaluate model performance. The following performance metrics were calculated from the confusion matrices: (1) F1 score-a weighted average of precision (positive predictive value), recall (sensitivity), specificity-the true negative rate, classification accuracy (CA)–the proportion of correctly classified cases.

The identified metabolomic signature was validated by generating a sPLS-DA (a supervised clustering ML approach that combines classification and parameter selection into one operation) with only metabolites that came up in three ML models of more. This analysis was performed using the R package mixOmics^77^ using the metabolomic data for the following comparisons: DC vs. HC; RRMS vs. HC; SPMS vs. HC; SPMS vs. RRMS; RRMS vs. DC and SPMS vs. DC.

To visualise how well individual metabolites (identified by six ML models or more) could discriminate SPMS from RRMS, compared to the metabolomic signature, area under the curve -receiver operating characteristic (AUC-ROC) curves were generated and based on the best performing ML model. RStudio 4.2.0 (The R Foundation, Vienna, Austria)87 were used for ML analysis.

#### Logistic regression with/without interactions

Less important features were shrunk to zero, using the least absolute shrinkage and selection operator (lasso) method which uses the absolute value of the co-efficient as a penalty. Tuning the regularisation variable lambda (λ), determines the strength of shrinkage. Lasso logistic regression (including with and without interactions) was conducted using the glmnet R package.^83^ All 111 metabolites were included. Categorical predictors were coded as dummy variables with the following treated as the reference class: smoking status–never smoked, sex–male and ethnicity–Caucasian/White. Age, baseline EDSS and BMI were treated as continuous variables. For the comparison RRMS vs. SPMS, an additional model where age and EDSS was excluded was generated for LR + I, to test whether metabolites alone can stratify RRMS and SPMS patients. The strength of shrinkage is determined by tuning the regularization variable lambda (λ). Ln(λ) was optimized using the R package and set to lambda = 0.059 and lambda = 0.065 for logistic regression with and without interactions respectively.

#### Ensemble methodology

To improve predictive performance of previous LR models, ensemble methods bootstrap aggregating (bagging) and boosting were utilised.^84^ **Bagging** involves training the model on each bootstrapped dataset. A bootstrapped dataset is made by randomly sampling patients from the original dataset with replacement i.e., a patient can appear more than once in the bootstrap dataset, however, the same number of patients must be used as the original dataset. This is then added to the ensemble, as an “individual”. Every “individual” that is added to the model has been trained on the same number but a different combination of patients. The aggregation process then involves collating each “individuals” assessment and the features i.e., metabolites that were voted for by the most individuals becomes the ensembles overall assessment. For each bootstrap dataset that is generated, a side product known as an out-of-bag dataset (patients not used within the bootstrap dataset) is generated and can be used for validation. **Boosting** trains each model to give more weight to previously misclassified patients from previous models, until all patients have been correctly predicted. The parameters for bagging and boosting were set to B = 100 and B = 1000, respectively with 10-fold cross-validation to prevent model overfitting. The ensemble models were created using the R Package caret^85^ and caTools packages.^86^

#### Random forest

RF is a machine-learning algorithm that assigns observations into classes (HC/RRMS/SPMS/DC) by creating thousands of decision trees (predicts the value of target variables by learning simple decision rules inferred from the features i.e., metabolites from the dataset), or a “forest”, and averaging the results. Only a small random sample of predictors are candidates for selection at each node, so the created trees are decorrelated. Importance was quantified by the Gini index, which represents the total variance across the two classes, quality of each split and the purity of each node. The values for mtry and ntree were tuned using the R Package caret.^85^ The parameters were set to mtry = 10, ntree = 10,000.

#### Support vector machine (SVM)

SVM is a supervised classification method which optimally separates data into two classes by creating a hyperplane. The radial basis function kernel was used, as this dataset was not linearly separable. Values for C, epsilon and gamma were tuned using the R Package 4.2.0.^99^ The parameters were set to C = 4.0, epsilon = 0.1, gamma = 0.01.

#### Neural network (NN)

NN uses the multi-layer perceptron algorithm with backpropagation which can learn both linear and non-linear models. NNs incorporate large numbers of processing nodes that are densely interconnected, similar to the human brain. These nodes are organised into layers-input, hidden and output. The output node/classification is affected by weights of values in hidden layers, these weights are adjusted when the model is trained for the best classification.

#### Model performance

10-fold cross-validation was used to evaluate model performance. The following performance metrics were calculated from the confusion matrices: (1) F1 score-a weighted average of precision (positive predictive value), recall (sensitivity), specificity-the true negative rate, classification accuracy (CA)–the proportion of correctly classified cases.

#### Sparse Partial Least Squares Discriminant Analysis (sPLS-DA)

sPLS-DA is a supervised clustering ML approach that combines classification and parameter selection into one operation. This analysis was performed using the R package mixOmics^77^ using the metabolomic data for the following comparisons: DC vs. HC; RRMS vs. HC; SPMS vs. HC; SPMS vs. RRMS; RRMS vs. DC and SPMS vs. DC. 10-fold cross validation with 50 repetitions was applied to prevent model overfitting. sPLS-DA models with different component numbers were assessed by 10-fold cross-validation using the balanced error rate to evaluate model performance. For the above comparisons, the number of components was selected, based on the combination that gave the lowest overall estimation error rate. These were selected as optimal and could give the best discriminatory performance for further analysis. The separation of the comparisons was presented by projecting the samples into the subspace constructed by component 1 and component 2. The top weighted features were selected and presented through variable loading plots. sPLS-DA results were visualised using the R package ggplot2.^87^

#### Metabolite scoring - Determining optimum cut points

Cut-point analysis was performed using the Youden index in conjunction with ROC analysis to determine optimum cut-offs or thresholds that can stratify RRMS from SPMS patients. This was done for each metabolite, identified by four ML models or more. The Youden index was calculated using the biomarker analysis tool in MetaboAnalyst 5.0.^21^

##### Building a clinical model based on serum metabolite expression to *discriminate* between RRMS vs. SPMS patients

AutoScore is a machine learning-based clinical score generator consisting of 6 modules (variable ranking, variable transformation, score derivation, model selection, score fine-tuning and model evaluation) for developing interpretable point-based scores.^22^ Compared to complex models, point-based scores are more explainable and interpretable and can be easily implemented and validated in clinical practice. The dataset on patients was divided into non-overlapping training (70%: 57 patients) and testing (30%: 24 patients) datasets. The training set was used to develop the clinical scoring model and the testing set was used to evaluate the best score/cut-off. Markers with a Youden index above 50% was used within the analysis. These variables were ranked by random forest (parameter – ntree = 100) and a parsimony plot used to identify the optimum number of variables to use within the clinical model. The parsimony plot considers both the no. of variables in each model against the model performance (measured by [AUC]) and chooses the optimum number of variables by balancing model complexity with predictive ability. To build the clinical model and produce scores, AutoScore uses multivariable logistic regression. Continuous variables were categorised, and quantiles was used to determine cut-off values of the data points. These initial scores generated by AutoScore were fine-tuned by using the guidance of cut-offs calculated using Youden Index to improve model interpretability. This fine-tuned model and optimum threshold (97 points) for stratifying RRMS from SPMS patients was evaluated on the test dataset.

#### Metabolite set enrichment analysis

Metabolite Set Enrichment Analysis was performed on the metabolomic signature identified by ML models (i.e., all metabolites identified by at least one ML model or more), sPLS-DA and forest plots using the KEGG and SMPDB database within MetaboAnalyst 5.0.^21^ This generated a report on over representation analysis. Metabolic pathways that were considered had p values<0.05.

#### Protein-protein interaction network analysis

To visualise protein-protein interactions, network analysis was performed in Cytoscape on genes taken from upregulated pathways.^81^ Mapped onto genes were FCs and colour-coded charts of top sub-pathways these genes belonged to. Enrichment maps were also generated to visualise the connection, significance, and clustering between sub-pathways under these upregulated GO and Reactome terms. Publication enrichment was performed to identify PubMed papers that had genes similar to the gene set from the selected pathways.^81^

#### Validation of gene expression pathways using independent datasets

To validate gene expression pathways, we used an independent study by Kihara et al.,^17^ who performed single nucleus RNA-seq on SPMS vs. RRMS patients. This was conducted on multiple cell types including, myeloid cells, lymphocytes, whole blood (myeloid and lymphocytes combined) and oligodendrocyte precursor cells. Multiple list enrichment (Metascape^79^) was used to compare pathways enriched across our dataset and Kihara et al., 2018. Only genes that had an FC < −1.5 or >+1.5 and FDR<0.05 were considered in the comparison.

#### Integration of transcriptomic and metabolomic data

##### Circos plots

Correlations between biological pathways identified by pathway enrichment analysis and the metabolomic signature that discriminates SPMS from RRMS were visualised using correlation circle plots.^88^ Pearson’s product moment correlation was calculated between the normalised count of pathway genes and metabolite levels.^89^ Only correlations with p < 0.01 or p < 0.05 were considered.

##### Gene-metabolite interaction network

Network analysis was performed in MetaboAnalyst using the gene-metabolite interaction network tool.^21^ This enables visualisation and exploration of interactions between functionally related genes within the DEG list and metabolites identified for the SPMS signature. It works by extracting human genes and chemical associations from STITCH to ensure only highly confident interactions are used. The log2FC was mapped onto each gene within the network. DisGeNET^82^ and literature search was used to identify associations between genes from the interaction network and autoimmune and neurological diseases.

#### Data visualisation

Volcano plots visualising differential gene expression were produced using EnhancedVolcano.^90^ RColorBrewer was used to generate color schemes and palettes for graphs.^91^ Circos plots were generated using Circlize which enables circular layout for chord plots to visualise large amounts of information.^88^ Boxplots were created in Ggpubr which also runs t-test and ANOVA analysis.^92^ Heatmaps to visualise expression levels of differentially expressed genes between groups were generated using Pheatmap^93^

### Quantification and statistical analysis

Metabolites identified by ML models were also analyzed using T tests, p values p < 0.05 were considered significant. Boxplots for individual metabolites were plotted and mean ± SD shown.

Genes which passed a threshold of both FDR<0.05 and FC±1.5 (log2FC: 0.585) were included differential gene expression analysis.

Pearson’s product moment correlation was calculated between the normalised count of pathway genes and metabolite levels.^89^ Only correlations with p < 0.01 and a Pearson coefficient >0.75 were plotted.

### Additional resources

RRMS patients were recruited as part of the ABIRISK study (Anti-Biopharmaceutical Immunization: Prediction and analysis of clinical relevance to minimize the risk of immunization in multiple sclerosis patients on interferon-beta treatment). This study is registered on the EU Clinical Trials Register https://www.clinicaltrialsregister.eu/ctr-search/trial/2012-005450-30/SE.

## Supplemental information

Document S1. Figures S1–S5 and Tables S1–S9 and S11

Table S10. Differentially expressed gene (DEG) lists for major enriched pathways; Metabolism of RNA, Cellular response to stress, Metabolism of lipids, Cellular respiration, Immune effector response, related to Figure 3 and Figure S3

Table S12. Differentially expressed genes (DEGs) identified by the gene-metabolite interaction network, related to Figure 4
