# Supplementary material for: Blood metabolomic and transcriptomic signatures stratify patient subgroups in multiple sclerosis according to disease severity
Source: iScience. 2024 Feb 15;27(3):109225. doi: 10.1016/j.isci.2024.109225 (PMC10907838; doi:10.1016/j.isci.2024.109225)
Supplement: Document S1. Figures S1–S5 and Tables S1–S9 and S11 [file mmc1.pdf]

iScience, Volume *27*

Supplemental information

Blood metabolomic and transcriptomic signatures stratify patient subgroups in multiple sclerosis according to disease severity

Alexandra E. Oppong, Leda Coelewij, Georgia Robertson, Lucia Martin-Gutierrez, Kirsty E. Waddington, Pierre Dönnes, Petra Nytrova, Rachel Farrell, Inés Pineda-Torra, and Elizabeth C. Jury

SUPPLEMENTAL INFORMATION

Figure S1: Study design Refer to Star Methods and Figures 1-4.

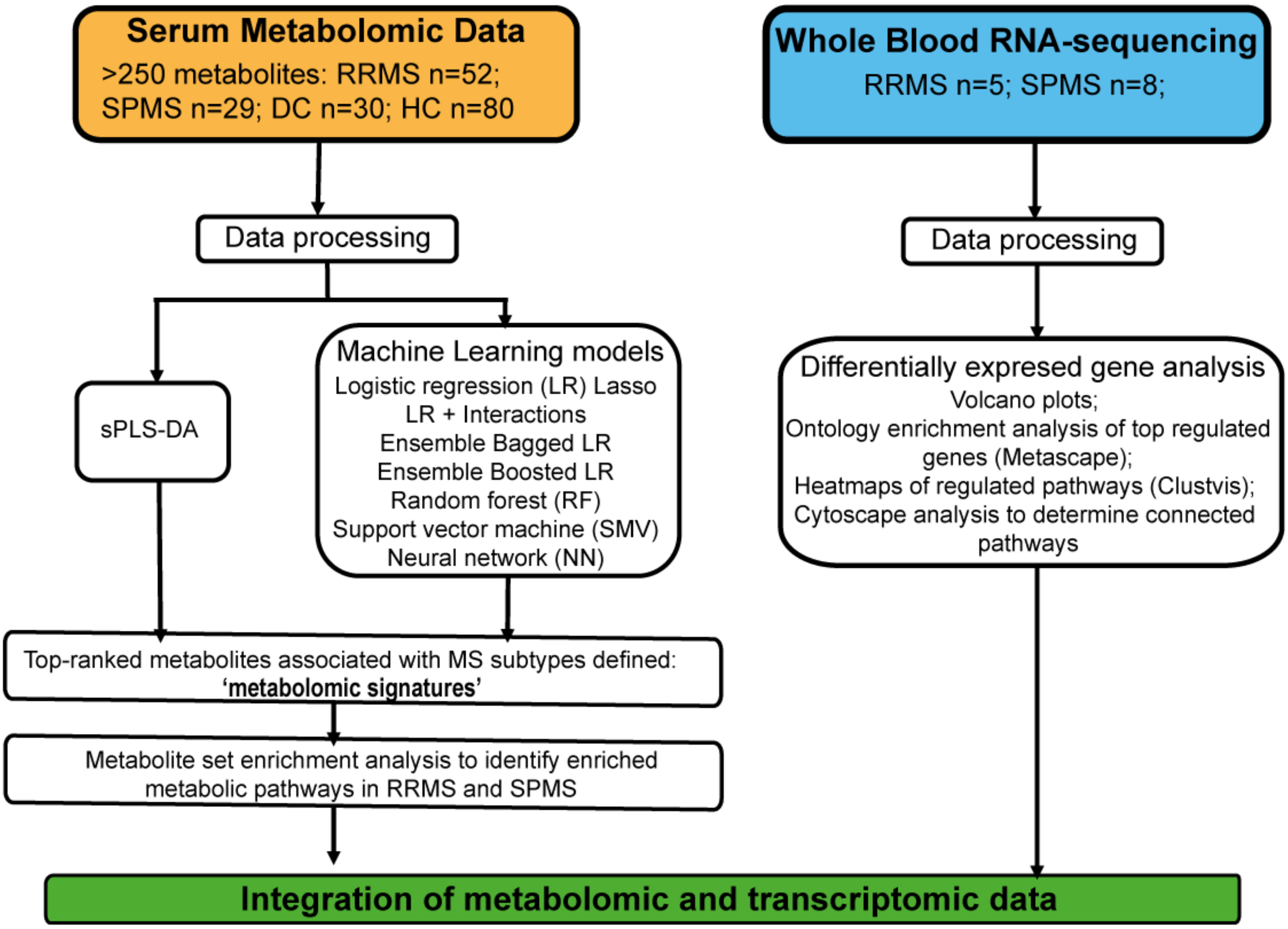

Figure S1: Study design. Refer to Star Methods and Figures 1-4. Pipeline of metabolomic and transcriptomic analyses down to final integration of metabolomic and transcriptomic signatures. sPLS-DA, sparse partial least squares-discriminant analysis. RNA-sequencing was performed on a subset of patients that also had matched metabolomic data. Data integration was performed on matched samples with both metabolomic and transcriptomic data.

Figure S2: sPLS-DA discriminates between patients with SPMS vs RRMS. Related to Figure 1, Table 2.

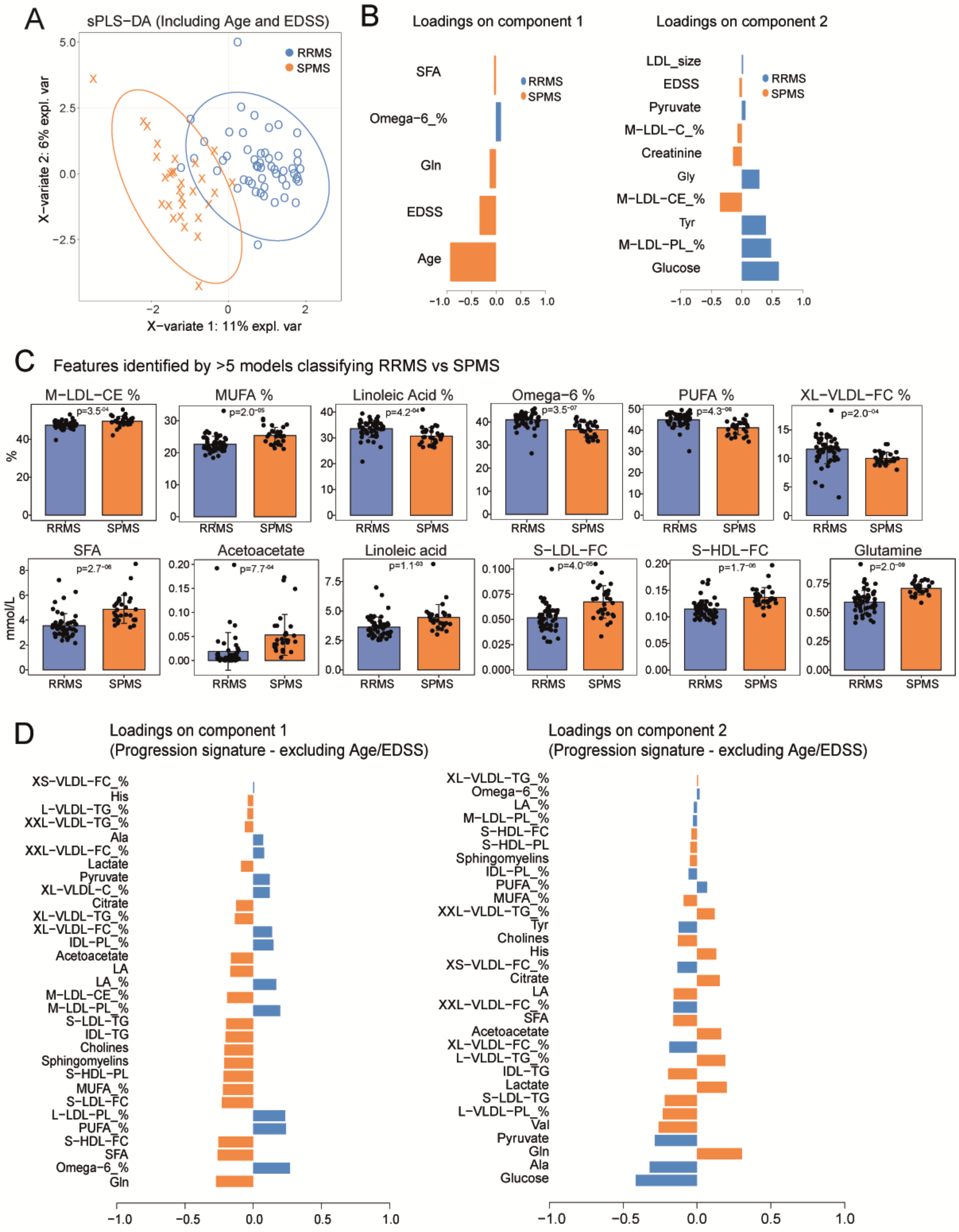

Figure S2: sPLS-DA discriminates between patients with SPMS vs RRMS. Related to Figure 1, Table 2. **(A-B)** sPLS-DA was performed using 7 components and 111 metabolite measurements and six clinical features (for model optimisation approach, refer to Supplementary Methods). (**A**) sPLS-DA plot showing level of separation of SPMS (orange) from RRMS (blue) patients. (**B**) The clinical features age and EDSS are higher in SPMS compared to RRMS

and provided the largest contributions on component 1, making these the most important features for clustering SPMS patients.The metabolites glucose, M-LDL-PL% and Tyr are higher in RRMS in comparison to SPMS and provided the largest contributions on component 2, making these the most important features for clustering RRMS patients.

(C) Bar charts showing relative expression of metabolites (% and mmol/L) in serum from patients with RRMS n=52 (blue) vs SPMS n=29 (orange). T-tests were performed to identify statistically significant differences. Plots show mean +/- SD.

(D) sPLS-DA was also used to validate the metabolomic signature discriminating between SPMS vs RRMS patients (See Figure 1C). Metabolites (identified by at least 3 machine learning models) contributing to loadings on components 1 and 2. The metabolites Gln, SFA, S-HDL-FC, Omega-6%, PUFA% and L-LDL-PL% provided the largest contributions on component 1 towards SPMS vs RRMS stratification. Glucose, Ala, Gln, Pyruvate, Val and L-VLDL-PL % provided the largest contributions on component 2 towards SPMS vs RRMS stratification. RRMS = blue and SPMS = orange.

Figure S3: Dysregulated pathways in SPMS vs RRMS. Related to Figure 3 and Tables S9-10

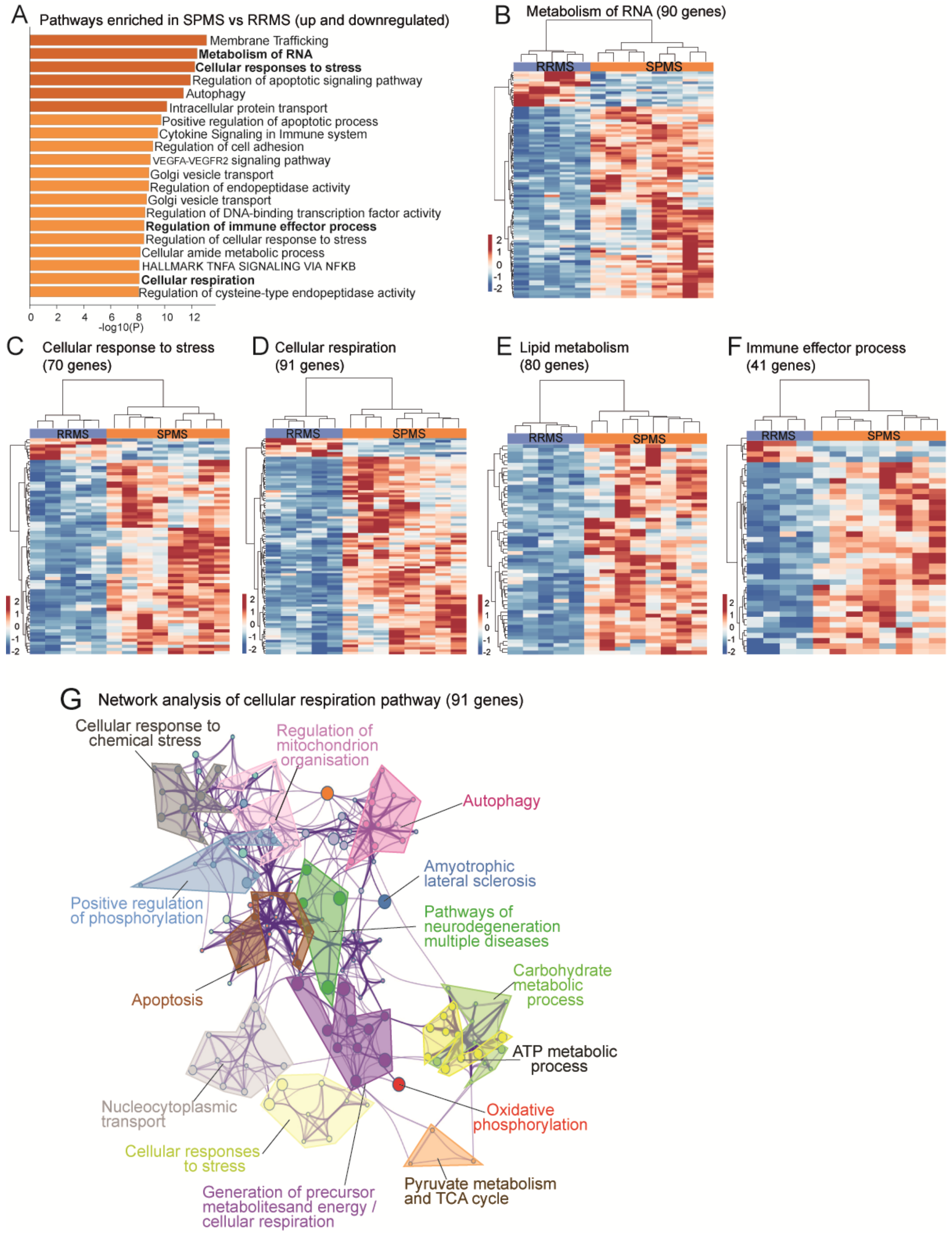

Supplementary Figure 3: Dysregulated pathways in SPMS vs RRMS. Related to Figure 3 and Tables S9-10. RNA-sequencing was performed on n=8 patients with SPMS and n=5 patients with RRMS followed by differential gene expression and pathway enrichment analysis**.** (**A**) Pathway enrichment analysis on all 1052 DEGs (combined up- and downregulated genes) analysed by Metascape to identify dysregulated pathways. Bar chart of top 20 significantly enriched pathways in SPMS patients (Gene Ontology (GO), Reactome, Hallmark, Wikipathways). Pathways are ranked by p-value. Pathways of interest are in black. (**B-F**) Heatmaps of normalised gene counts in the (B) 'Metabolism of RNA', (C) 'Cellular responses to stress', (D) 'Cellular respiration' (E) 'Metabolism of Lipids', (F) 'Immune effector process' pathways. See Table S8 for DEG lists. **(G)** Network diagram illustrates pathways associated with the genes significantly up and downregulated in the cellular respiration pathway.

Figure S4: Correlation between DEGs and metabolites. Related to Table S11.

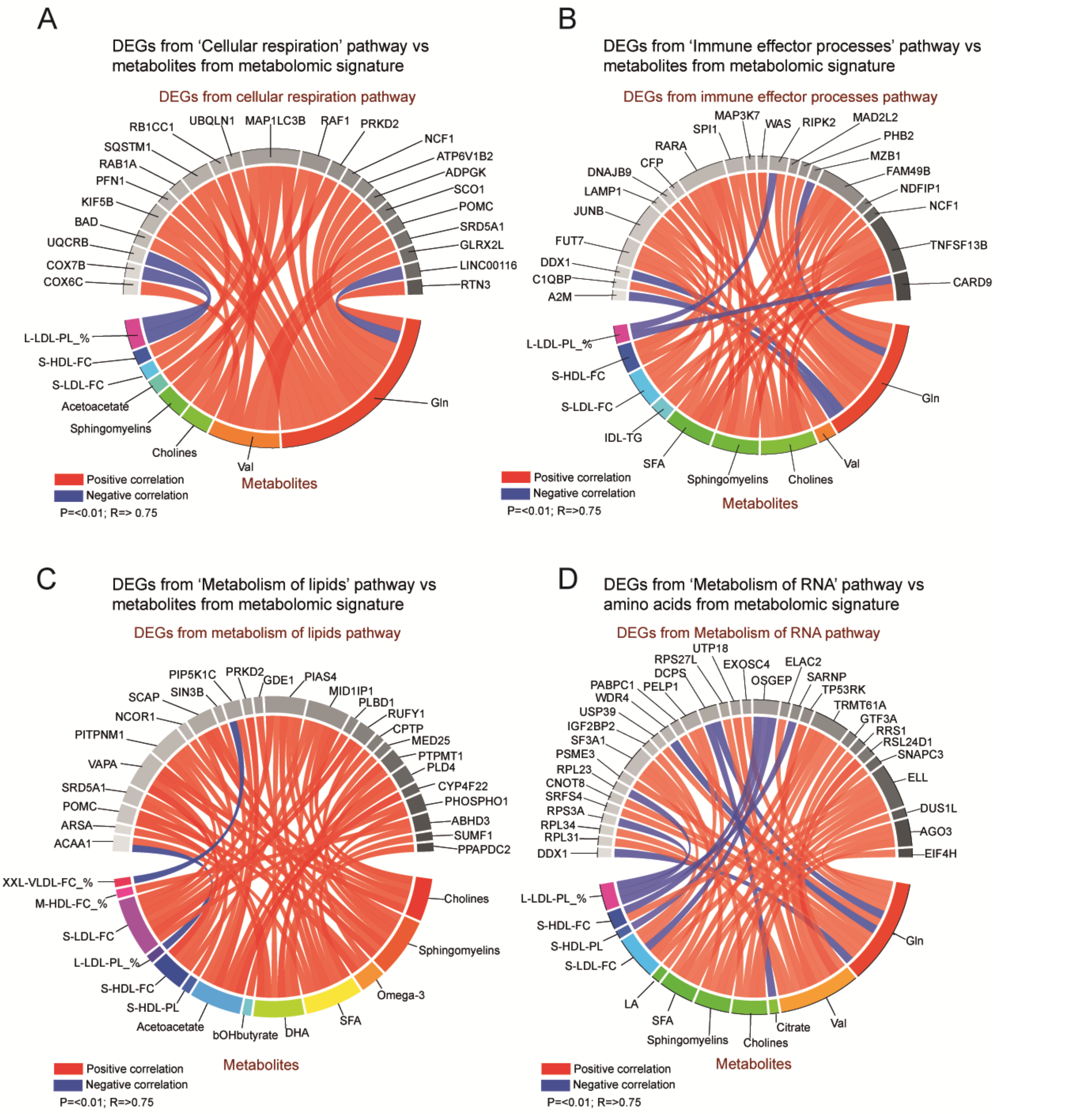

Figure S4: Correlation between DEGs and metabolites. Related to Table S11. Chord plots showing significant correlations between gene counts from DEGs in significantly enriched pathways (SPMS vs RRMS) and metabolites identified in Figure 1B. (**A**) Significant correlations between DEGs in cellular respiration pathway and metabolomic marker concentrations. Only significant associations (p-value <0.01) with a Pearson coefficient >0.75 are plotted. (**B**) Significant correlations between DEGs in 'regulation of immune effector process' pathway and metabolomic marker concentrations. Only significant associations (p-value <0.01) with a Pearson coefficient >0.75 are plotted; **(C)** Significant correlations between DEGs in the 'metabolism of lipids' pathway and metabolomic marker concentrations. Only significant associations p-value <0.01 Pearson coefficient >0.75 were plotted. (**D**) Significant correlations between DEGs in the Metabolism of RNA' pathway and metabolomic marker concentrations. Only significant associations p-value <0.01, with a Pearson coefficient >0.75 were plotted. Red lines = positive correlation; blue lines = negative correlations.

Figure S5: Summary of potential metabolic changes in peripheral blood from people with SPMS vs RRMS. Related to Figure 4.

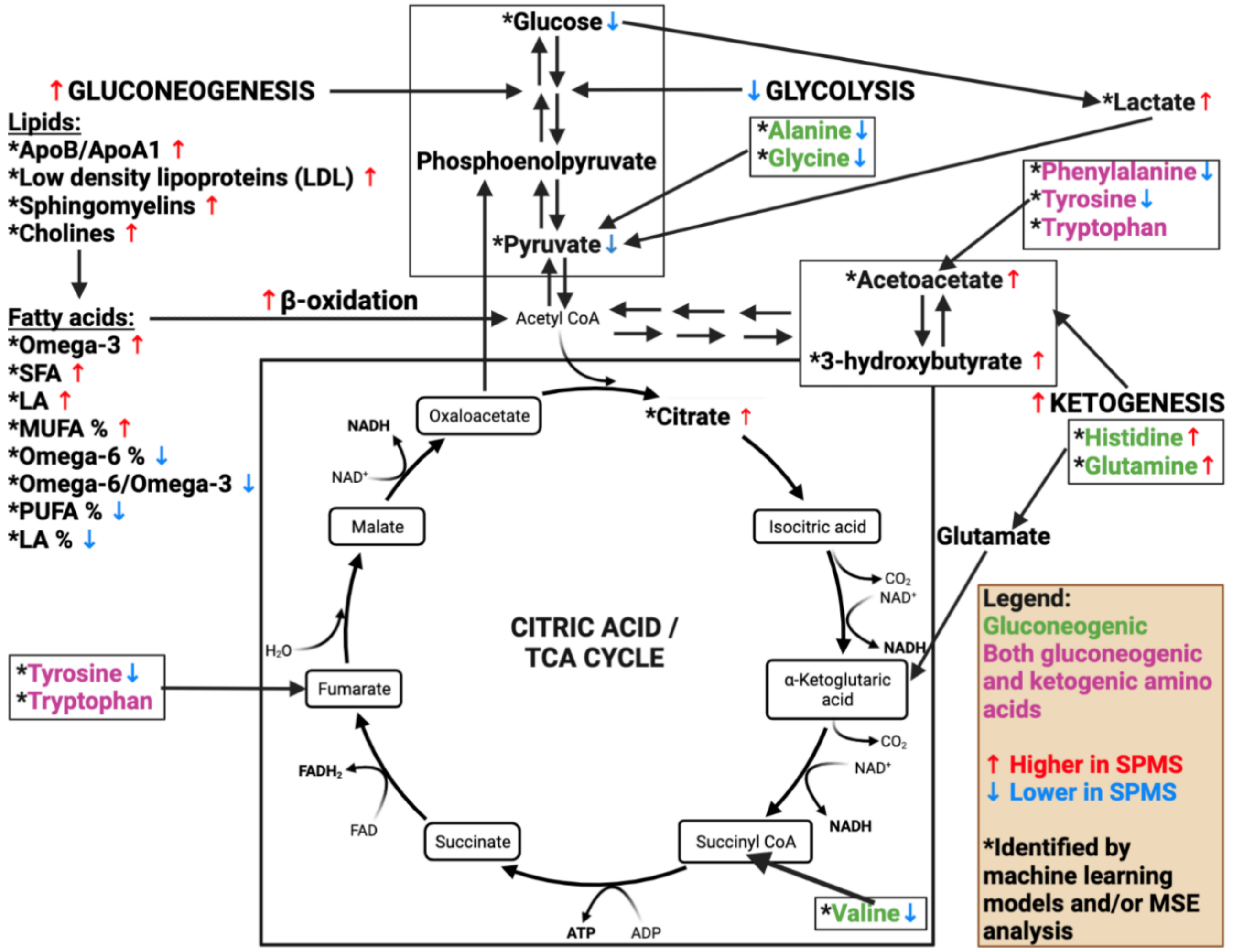

Figure S5. Summary of peripheral blood metabolic pathways differentially regulated in patients with SPMS vs RRMS. Related to Figure 4. The results suggest a potential metabolic switch from glycolysis towards increased gluconeogenesis and ketogenesis, supported by fatty acid β-oxidation and conversion of ketogenic and gluconeogenic amino acids, phenylalanine, and tyrosine in patients with SPMS compared to those with RRMS.

Table S1: Cohort characteristics: Refer to Star Methods.

Cohort characteristics (metabolomics)					
**Clinical features**		**RRMS (n=52)**	**SPMS (n=29)**	**Healthy Controls (n=80)**	**Disease Controls (n=30)**
**Sex, N (%)**	Female	37 (71)	18 (62)	56 (70)	25 (83)
	Male	15 (29)	11 (38)	24 (30)	5 (17)
**Age, years**	Median (Range)	34 (19-59)	54 (35-78)	36 (19-76)	48 (23-66)
**Ethnicity, N (%)**	White	52 (100)	26 (90)	54 (68)	0 (0)
	Black/Caribbean	0 (0)	2 (7)	7 (9)	0 (0)
	Asian/Indian	0 (0)	0	11 (14)	0 (0)
	Mixed/Other	0 (0)	1 (3)	1 (1)	0 (0)
	NA	0 (0)	0	7 (9)	30 (100)
***EDSS Score**	Median (Range)	2 (0-4)	4 (0-8)	NA	NA
**BMI**	Median (Range)	23.7 (15.2-44.6)	25.8 (16.0-36.6)	-	-
**Smoking Status, N (%)**	Never smoked	32 (61.5)	12 (41)	-	-
	Quit Smoking	8 (15.4)	15 (52)	-	-
	Current smoker	12 (23.1)	2 (7)	-	-
	NA	0 (0)	0(0)	80 (100)	30 (100)

Cohort characteristics (RNA-sequencing)				
**Clinical Features**		**RRMS (n=5)**	**SPMS (n=8)**	**P-value**
**Sex n (%)**	Female	3 (60)	6 (75)	0.5686*
	Male	2 (40)	2 (25)	
**Age (years)**	Median (IQR)	34 (3)	57.5 (11)	0.006691#
**Ethnicity n (%)**	White	5 (100)	7 (87.5)	0.4106
	Mixed/Other	0 (0)	1 (12.5)	
***EDSS**	Median (IQR)	2.0 (0.5)	6 (0)	0.03142
**BMI**	Median (IQR)	24.3 (1.9)	28.1 (7.5)	0.63892
**Smoking n (%)**	Non-smoker	2 (40)	3 (37.5)	0.70921
	Quit smoking	3 (60)	4 (50)	
	Current smoker	0 (0)	0 (0)	
	Not disclosed	0 (0)	1 (12.5)	
**DMT n (%)**	IFNβ-1a	3 (60)	0 (0)	N/A
	IFNβ-1b	2 (40)	0 (0)	
	None	0 (0)	8 (100)	

Table S1. Cohort characteristics: Refer to Star Methods. All patients with Relapsing-remitting multiple sclerosis (RRMS) were recruited before treatment with first disease modifying therapy. Patients with secondary progressive multiple sclerosis (SPMS): at time of blood sampling n=27 were on no disease modifying therapy (DMT); n=1 patient had recived Ocrelizumab; n=1 patient had received Teriflunomide. Patients with neuromyelitis optica were included as disease controls (DCs). Metabolomics was performed on the total cohort (RRMS n=53; SPMS n=27, HC, n=80; DC, n=30). Transcriptomics was performed on a smaller subset of MS patients (RRMS, n=5; SPMS, n=8). All patients in the transcriptomics (RNA-sequencing) cohort were also included in the metabolomic cohort and had matched metabolomic analysis.

Table S2: List of metabolites and abbreviations (Nightingale Health)

Abbreviation	Full name
Total-C	Total cholesterol
non-HDL-C	Total cholesterol minus HDL-C
Remnant-C	Remnant cholesterol (non-HDL, non-LDL -cholesterol)
VLDL-C	VLDL cholesterol
Clinical LDL-C	Clinical LDL cholesterol
LDL-C	LDL cholesterol
HDL-C	HDL cholesterol
Total-TG	Total triglycerides
VLDL-TG	Triglycerides in VLDL
LDL-TG	Triglycerides in LDL
HDL-TG	Triglycerides in HDL
Total-PL	Total phospholipids in lipoprotein particles
VLDL-PL	Phospholipids in VLDL
LDL-PL	Phospholipids in LDL
HDL-PL	Phospholipids in HDL
Total-CE	Total esterified cholesterol
VLDL-CE	Cholesteryl esters in VLDL
LDL-CE	Cholesteryl esters in LDL
HDL-CE	Cholesteryl esters in HDL
Total-FC	Total free cholesterol
VLDL-FC	Free cholesterol in VLDL
LDL-FC	Free cholesterol in LDL
HDL-FC	Free cholesterol in HDL
Total-L	Total lipids in lipoprotein particles
VLDL-L	Total lipids in VLDL
LDL-L	Total lipids in LDL
HDL-L	Total lipids in HDL
Total-P	Total concentration of lipoprotein particles
VLDL-P	Concentration of VLDL particles
LDL-P	Concentration of LDL particles
HDL-P	Concentration of HDL particles
VLDL size	Average diameter for VLDL particles
LDL size	Average diameter for LDL particles
HDL size	Average diameter for HDL particles
Phosphoglyc	Phosphoglycerides
TG/PG	Ratio of triglycerides to phosphoglycerides
Cholines	Total cholines
Phosphatidylc	Phosphatidylcholines
Sphingomyelins	Sphingomyelins
ApoB	Apolipoprotein B
ApoA1	Apolipoprotein A1
ApoB/ApoA1	Ratio of apolipoprotein B to apolipoprotein A1
Total-FA	Total fatty acids
Unsaturation	Degree of unsaturation
Omega-3	Omega-3 fatty acids
Omega-6	Omega-6 fatty acids
PUFA	Polyunsaturated fatty acids
MUFA	Monounsaturated fatty acids
SFA	Saturated fatty acids
LA	Linoleic acid
DHA	Docosahexaenoic acid
Omega-3 %	Ratio of omega-3 fatty acids to total fatty acids
Omega-6 %	Ratio of omega-6 fatty acids to total fatty acids
PUFA %	Ratio of polyunsaturated fatty acids to total fatty acids
MUFA %	Ratio of monounsaturated fatty acids to total fatty acids
SFA %	Ratio of saturated fatty acids to total fatty acids
LA %	Ratio of linoleic acid to total fatty acids
DHA %	Ratio of docosahexaenoic acid to total fatty acids
PUFA/MUFA	Ratio of polyunsaturated fatty acids to monounsaturated fatty acids
Omega-6/Omega-3	Ratio of omega-6 fatty acids to omega-3 fatty acids
Ala	Alanine
Gln	Glutamine
Gly	Glycine
His	Histidine
Total BCAA	Total concentration of branched-chain amino acids (leucine + isoleucine + valine)
Ile	Isoleucine
Leu	Leucine
Val	Valine
Phe	Phenylalanine
Tyr	Tyrosine
Glucose	Glucose
Lactate	Lactate
Pyruvate	Pyruvate
Citrate	Citrate
Glycerol	Glycerol
bOHbutyrate	3-Hydroxybutyrate
Acetate	Acetate
Acetoacetate	Acetoacetate
Acetone	Acetone
Creatinine	Creatinine
Albumin	Albumin
GlycA	Glycoprotein acetyls
XXL-VLDL-P	Concentration of chylomicrons and extremely large VLDL particles
XXL-VLDL-L	Total lipids in chylomicrons and extremely large VLDL
XXL-VLDL-PL	Phospholipids in chylomicrons and extremely large VLDL
XXL-VLDL-C	Cholesterol in chylomicrons and extremely large VLDL
XXL-VLDL-CE	Cholesteryl esters in chylomicrons and extremely large VLDL
XXL-VLDL-FC	Free cholesterol in chylomicrons and extremely large VLDL
XXL-VLDL-TG	Triglycerides in chylomicrons and extremely large VLDL
XL-VLDL-P	Concentration of very large VLDL particles
XL-VLDL-L	Total lipids in very large VLDL

XL-VLDL-PL	Phospholipids in very large VLDL
XL-VLDL-C	Cholesterol in very large VLDL
XL-VLDL-CE	Cholesteryl esters in very large VLDL
XL-VLDL-FC	Free cholesterol in very large VLDL
XL-VLDL-TG	Triglycerides in very large VLDL
L-VLDL-P	Concentration of large VLDL particles
L-VLDL-L	Total lipids in large VLDL
L-VLDL-PL	Phospholipids in large VLDL
L-VLDL-C	Cholesterol in large VLDL
L-VLDL-CE	Cholesteryl esters in large VLDL
L-VLDL-FC	Free cholesterol in large VLDL
L-VLDL-TG	Triglycerides in large VLDL
M-VLDL-P	Concentration of medium VLDL particles
M-VLDL-L	Total lipids in medium VLDL
M-VLDL-PL	Phospholipids in medium VLDL
M-VLDL-C	Cholesterol in medium VLDL
M-VLDL-CE	Cholesteryl esters in medium VLDL
M-VLDL-FC	Free cholesterol in medium VLDL
M-VLDL-TG	Triglycerides in medium VLDL
S-VLDL-P	Concentration of small VLDL particles
S-VLDL-L	Total lipids in small VLDL
S-VLDL-PL	Phospholipids in small VLDL
S-VLDL-C	Cholesterol in small VLDL
S-VLDL-CE	Cholesteryl esters in small VLDL
S-VLDL-FC	Free cholesterol in small VLDL
S-VLDL-TG	Triglycerides in small VLDL
XS-VLDL-P	Concentration of very small VLDL particles
XS-VLDL-L	Total lipids in very small VLDL
XS-VLDL-PL	Phospholipids in very small VLDL
XS-VLDL-C	Cholesterol in very small VLDL
XS-VLDL-CE	Cholesteryl esters in very small VLDL
XS-VLDL-FC	Free cholesterol in very small VLDL
XS-VLDL-TG	Triglycerides in very small VLDL
IDL-P	Concentration of IDL particles
IDL-L	Total lipids in IDL
IDL-PL	Phospholipids in IDL
IDL-C	Cholesterol in IDL
IDL-CE	Cholesteryl esters in IDL
IDL-FC	Free cholesterol in IDL
IDL-TG	Triglycerides in IDL
L-LDL-P	Concentration of large LDL particles
L-LDL-L	Total lipids in large LDL
L-LDL-PL	Phospholipids in large LDL
L-LDL-C	Cholesterol in large LDL
L-LDL-CE	Cholesteryl esters in large LDL
L-LDL-FC	Free cholesterol in large LDL
L-LDL-TG	Triglycerides in large LDL
M-LDL-P	Concentration of medium LDL particles
M-LDL-L	Total lipids in medium LDL
M-LDL-PL	Phospholipids in medium LDL
M-LDL-C	Cholesterol in medium LDL
M-LDL-CE	Cholesteryl esters in medium LDL
M-LDL-FC	Free cholesterol in medium LDL
M-LDL-TG	Triglycerides in medium LDL
S-LDL-P	Concentration of small LDL particles
S-LDL-L	Total lipids in small LDL
S-LDL-PL	Phospholipids in small LDL
S-LDL-C	Cholesterol in small LDL
S-LDL-CE	Cholesteryl esters in small LDL
S-LDL-FC	Free cholesterol in small LDL
S-LDL-TG	Triglycerides in small LDL
XL-HDL-P	Concentration of very large HDL particles
XL-HDL-L	Total lipids in very large HDL
XL-HDL-PL	Phospholipids in very large HDL
XL-HDL-C	Cholesterol in very large HDL
XL-HDL-CE	Cholesteryl esters in very large HDL
XL-HDL-FC	Free cholesterol in very large HDL
XL-HDL-TG	Triglycerides in very large HDL
L-HDL-P	Concentration of large HDL particles
L-HDL-L	Total lipids in large HDL
L-HDL-PL	Phospholipids in large HDL
L-HDL-C	Cholesterol in large HDL
L-HDL-CE	Cholesteryl esters in large HDL
L-HDL-FC	Free cholesterol in large HDL
L-HDL-TG	Triglycerides in large HDL
M-HDL-P	Concentration of medium HDL particles
M-HDL-L	Total lipids in medium HDL
M-HDL-PL	Phospholipids in medium HDL
M-HDL-C	Cholesterol in medium HDL
M-HDL-CE	Cholesteryl esters in medium HDL
M-HDL-FC	Free cholesterol in medium HDL
M-HDL-TG	Triglycerides in medium HDL
S-HDL-P	Concentration of small HDL particles
S-HDL-L	Total lipids in small HDL
S-HDL-PL	Phospholipids in small HDL
S-HDL-C	Cholesterol in small HDL
S-HDL-CE	Cholesteryl esters in small HDL
S-HDL-FC	Free cholesterol in small HDL
S-HDL-TG	Triglycerides in small HDL
XXL-VLDL-PL %	Phospholipids to total lipids ratio in chylomicrons and extremely large VLDL
XXL-VLDL-C %	Cholesterol to total lipids ratio in chylomicrons and extremely large VLDL
XXL-VLDL-CE %	Cholesteryl esters to total lipids ratio in chylomicrons and extremely large VLDL
XXL-VLDL-FC %	Free cholesterol to total lipids ratio in chylomicrons and extremely large VLDL
XXL-VLDL-TG %	Triglycerides to total lipids ratio in chylomicrons and extremely large VLDL
XL-VLDL-PL %	Phospholipids to total lipids ratio in very large VLDL
XL-VLDL-C %	Cholesterol to total lipids ratio in very large VLDL

XL-VLDL-CE %	Cholesteryl esters to total lipids ratio in very large VLDL
XL-VLDL-FC %	Free cholesterol to total lipids ratio in very large VLDL
XL-VLDL-TG %	Triglycerides to total lipids ratio in very large VLDL
L-VLDL-PL %	Phospholipids to total lipids ratio in large VLDL
L-VLDL-C %	Cholesterol to total lipids ratio in large VLDL
L-VLDL-CE %	Cholesteryl esters to total lipids ratio in large VLDL
L-VLDL-FC %	Free cholesterol to total lipids ratio in large VLDL
L-VLDL-TG %	Triglycerides to total lipids ratio in large VLDL
M-VLDL-PL %	Phospholipids to total lipids ratio in medium VLDL
M-VLDL-C %	Cholesterol to total lipids ratio in medium VLDL
M-VLDL-CE %	Cholesteryl esters to total lipids ratio in medium VLDL
M-VLDL-FC %	Free cholesterol to total lipids ratio in medium VLDL
M-VLDL-TG %	Triglycerides to total lipids ratio in medium VLDL
S-VLDL-PL %	Phospholipids to total lipids ratio in small VLDL
S-VLDL-C %	Cholesterol to total lipids ratio in small VLDL
S-VLDL-CE %	Cholesteryl esters to total lipids ratio in small VLDL
S-VLDL-FC %	Free cholesterol to total lipids ratio in small VLDL
S-VLDL-TG %	Triglycerides to total lipids ratio in small VLDL
XS-VLDL-PL %	Phospholipids to total lipids ratio in very small VLDL
XS-VLDL-C %	Cholesterol to total lipids ratio in very small VLDL
XS-VLDL-CE %	Cholesteryl esters to total lipids ratio in very small VLDL
XS-VLDL-FC %	Free cholesterol to total lipids ratio in very small VLDL
XS-VLDL-TG %	Triglycerides to total lipids ratio in very small VLDL
IDL-PL %	Phospholipids to total lipids ratio in IDL
IDL-C %	Cholesterol to total lipids ratio in IDL
IDL-CE %	Cholesteryl esters to total lipids ratio in IDL
IDL-FC %	Free cholesterol to total lipids ratio in IDL
IDL-TG %	Triglycerides to total lipids ratio in IDL
L-LDL-PL %	Phospholipids to total lipids ratio in large LDL
L-LDL-C %	Cholesterol to total lipids ratio in large LDL
L-LDL-CE %	Cholesteryl esters to total lipids ratio in large LDL
L-LDL-FC %	Free cholesterol to total lipids ratio in large LDL

L-LDL-TG %	Triglycerides to total lipids ratio in large LDL
M-LDL-PL %	Phospholipids to total lipids ratio in medium LDL
M-LDL-C %	Cholesterol to total lipids ratio in medium LDL
M-LDL-CE %	Cholesteryl esters to total lipids ratio in medium LDL
M-LDL-FC %	Free cholesterol to total lipids ratio in medium LDL
M-LDL-TG %	Triglycerides to total lipids ratio in medium LDL
S-LDL-PL %	Phospholipids to total lipids ratio in small LDL
S-LDL-C %	Cholesterol to total lipids ratio in small LDL
S-LDL-CE %	Cholesteryl esters to total lipids ratio in small LDL
S-LDL-FC %	Free cholesterol to total lipids ratio in small LDL
S-LDL-TG %	Triglycerides to total lipids ratio in small LDL
XL-HDL-PL %	Phospholipids to total lipids ratio in very large HDL
XL-HDL-C %	Cholesterol to total lipids ratio in very large HDL
XL-HDL-CE %	Cholesteryl esters to total lipids ratio in very large HDL
XL-HDL-FC %	Free cholesterol to total lipids ratio in very large HDL
XL-HDL-TG %	Triglycerides to total lipids ratio in very large HDL
L-HDL-PL %	Phospholipids to total lipids ratio in large HDL
L-HDL-C %	Cholesterol to total lipids ratio in large HDL
L-HDL-CE %	Cholesteryl esters to total lipids ratio in large HDL
L-HDL-FC %	Free cholesterol to total lipids ratio in large HDL
L-HDL-TG %	Triglycerides to total lipids ratio in large HDL
M-HDL-PL %	Phospholipids to total lipids ratio in medium HDL
M-HDL-C %	Cholesterol to total lipids ratio in medium HDL
M-HDL-CE %	Cholesteryl esters to total lipids ratio in medium HDL
M-HDL-FC %	Free cholesterol to total lipids ratio in medium HDL
M-HDL-TG %	Triglycerides to total lipids ratio in medium HDL
S-HDL-PL %	Phospholipids to total lipids ratio in small HDL
S-HDL-C %	Cholesterol to total lipids ratio in small HDL
S-HDL-CE %	Cholesteryl esters to total lipids ratio in small HDL
S-HDL-FC %	Free cholesterol to total lipids ratio in small HDL
S-HDL-TG %	Triglycerides to total lipids ratio in small HDL

Table S3. Table showing all features identified by one or more models classifying HCs vs RRMS and HCs vs SPMS (Related to Table 1).

Top features HC vs RRMS

Features	LR	LR+I	Bagged LR	Boosted LR	RF	SVM	NN
**His**	■	■	■	■	■	■	■
**XS-VLDL-FC %**	■	■	■	■	■	■	■
**Acetoacetate**	■	■	■	■	■	■	
**LDL size**	■	■	■	■		■	■
**M-HDL-PL**	■		■	■	■	■	
**S-HDL-FC**		■	■	■	■	■	
**XL-VLDL-FC %**		■	■	■	■	■	
**XL-VLDL-TG %**		■	■	■	■	■	
**Cholines**		■	■	■	■	■	
**L-VLDL-TG %**		■	■	■	■	■	
**XS-VLDL-PL %**		■	■	■	■	■	
**S-VLDL-CE %**		■	■	■		■	
**Unsaturation**		■	■	■		■	
**M-VLDL-PL %**			■	■	■	■	
**S-HDL-PL**			■	■	■	■	
**L-VLDL-C %**			■	■	■	■	
**S-HDL-CE**			■	■	■	■	
**IDL-PL %**	■		■	■		■	
**Pyruvate**	■	■			■		■
**Acetone**	■	■			■		■
**Ile**	■	■			■		■
**Acetate**		■			■		■
**XXL-VLDL-FC %**	■	■					■
**Phe**	■	■					■
**Albumin**	■	■					■
**SFA %**	■	■					■
**Omega-6/Omega-3**	■	■					■
**S-LDL-C %**	■	■					■
**PUFA %**			■	■		■	
**SFA**			■	■		■	
bOHbutyrate	■	■					
Citrate	■	■					
Ethnicity	■						■
XXL-VLDL-CE %	■						■
Tyr	■				■		
S-VLDL-CE %		■			■		
Ala		■					■
Creatinine		■					■
M-LDL-FC %		■					■
S-LDL-FC %		■					■
Lactate		■					
XXL-VLDL-PL %		■					
XXL-VLDL-C %		■					
XL-VLDL-PL %		■					
XL-VLDL-C %		■					
XL-VLDL-CE %		■					
L-LDL-FC %		■					
XL-HDL-PL %		■					
XXL-VLDL-TG		■					
XXL-VLDL-TG %		■					
XS-VLDL-PL		■					
L-HDL-TG %		■					
L-LDL-CE %		■					
L-HDL-PL %		■					
S-HDL-PL %		■					
LA %		■					
Omega-3		■					
Omega-3 %		■					
M-LDL-PL %	■						
Sex							■

Top features HC vs SPMS

Features	LR	LR+I	Bagged LR	Boosted LR	RF	SVM	NN
**Age**	■	■	■	■	■	■	■
**Acetone**	■	■	■	■	■	■	■
**Gln**	■	■	■	■	■	■	■
**LA %**	■	■	■	■	■	■	■
**Tyr**	■	■	■	■	■	■	■
**Omega-3**	■	■	■	■		■	■
**bOHbutyrate**	■		■	■	■	■	
**Acetoacetate**		■	■	■	■	■	
**Omega-6 %**			■	■	■	■	
**SFA**			■	■	■	■	
**XS-VLDL-CE**			■	■	■	■	
**S-LDL-FC**			■	■	■	■	
**L-LDL-P**			■	■	■	■	
**IDL-P**			■	■	■	■	
**ApoB/ApoA1**	■		■	■		■	
**IDL-FC**		■	■	■		■	
**Glucose**	■	■			■		■
**Lactate**	■	■			■		■
**Acetate**	■	■			■		■
**His**	■	■					■
**S-LDL-PL %**	■	■					■
**Albumin**	■	■			■		
**Ala**		■			■		■
**Val**		■			■		■
**S-VLDL-CE**			■	■		■	
**XS-VLDL-PL**			■	■		■	
**M-VLDL-CE**			■	■		■	
**IDL-TG**			■	■		■	
XL-VLDL-PL %	■	■					
Omega-3 %	■						■
LDL size		■					■
XXL-VLDL-CE %		■					■
XXL-VLDL-PL %		■					■
IDL-FC %		■					■
VLDL size		■					
SFA %		■					
PUFA/MUFA		■					
XXLVLDL-FC %		■					
L-LDL-CE %		■					
M-LDL-C %		■					
XL-HDL-FC %		■					
GlycA		■					
L-VLDL-FC %		■					
XS-VLDL-PL %		■					
XS-VLDL-FC %		■					
IDL-PL %		■					
Citrate		■					
Creatinine		■					
XL-HDL-TG		■					
XL-VLDL-TG %		■					
L-LDL-PL %		■					
S-LDL-CE %		■					
M-HDL-PL %		■					
L-VLDL-C %					■		
S-LDL-C %							■
Ethnicity							■

Red= features identified in >6 models
Bold= features identified in >3 models
LR: Logistic Regression
LR + Interactions (I)
RF: Random Forest
SVM: Support Vector Machine
NN: Neural Network

Table S4. Table showing all features identified by one or more models classifying DCs vs RRMS and DC vs SPMS. Related to Table 1

Top features DC vs RRMS — Models

Features	LR	LR+I	Bagged LR	Boosted LR	RF	SVM	NN
**Age**	■	■	■	■	■	■	■
**Acetone**	■	■	■	■	■	■	■
**Gly**	■	■	■	■	■	■	■
**XL-VLDL-CE %**	■	■	■	■	■	■	■
**IDL-FC %**	■	■	■	■	■	■	■
**Acetoacetate**	■	■	■	■	■	■	
**XL-VLDL-TG %**			■	■	■	■	■
**L-VLDL-C %**			■	■	■	■	
**SFA**			■	■	■	■	
**IDL-TG**			■	■	■	■	
**PUFA %**			■	■	■	■	
**XL-HDL-TG**			■	■	■	■	
**XL-HDL-TG %**			■	■	■	■	
**VLDL size**			■	■	■	■	
**S-VLDL-CE %**			■	■	■	■	
**XXL-VLDL-TG**		■	■	■		■	
**TG-PG**		■	■	■		■	
**MUFA %**			■	■		■	
**M-LDL-CE %**			■	■		■	
**M-LDL-FC %**			■	■		■	
**bOHbutyrate**	■	■					■
**Ala**	■	■					■
**Gln**	■	■					■
**Glycerol**	■	■					■
**XXL-VLDL-CE %**	■	■					■
**M-LDL-PL %**	■	■					■
**XL-HDL-PL %**	■	■					
**S-HDL-PL %**	■	■					■
Sex	■						■
LA		■			■		
Creatinine		■					■
Isoleucine		■					■
XS-VLDL-PL %		■					■
Leu		■					■
TG-PG %					■		
L-VLDL-FC %					■		
Sphingomyelins					■		
Phe					■		
XL-HDL-CE %							■
DHA %							■
ApoB/ApoA1		■					
Omega-3		■					
DHA		■					
Omega-3 %		■					
LA %		■					
Omega-6/Omega-3		■					
Pyruvate		■					
Acetate		■					
S-LDL-FC		■					
XXL-VLDL-C %		■					
IDL-PL %		■					
XXL-VLDL-TG %		■					
Lactate		■					
L-HDL-PL %		■					
XL-HDL-FC %		■					
XL-VLDL-PL %		■					
L-HDL-TG %		■					
M-HDL-C %		■					

Top features DC vs SPMS — Models

Features	LR	LR+I	Bagged LR	Boosted LR	RF	SVM	NN
**Acetone**	■	■	■	■	■	■	■
**Pyruvate**	■	■	■	■	■	■	■
**Lactate**	■	■	■	■	■	■	■
**Phe**	■	■	■	■	■	■	
**DHA**	■	■	■	■	■	■	
**Glutamine**	■		■	■	■	■	■
**Leu**	■		■	■	■	■	■
**His**	■		■	■	■	■	■
**XL-HDL-CE %**	■		■	■	■	■	■
**S-HDL-PL**	■		■	■	■	■	
**XL-VLDL-FC %**	■		■	■	■	■	
**Acetoacetate**			■	■	■	■	■
**XL-HDL-PL %**			■	■	■	■	■
**XL-HDL-C %**		■	■	■		■	■
**bOHbuytrate**			■	■	■	■	
**Leu**			■	■	■	■	
**Glucose**			■	■	■	■	
**L-LDL-CE %**			■	■	■	■	
**Gly**		■	■	■		■	
**Citrate**			■	■		■	
Tyr		■					■
GlycA		■					■
Sphingomyelins		■					■
PUFA/MUFA		■					■
IDL-FC %		■					■
L-VLDL-TG %		■					■
XXL-VLDL-CE %		■					■
XL-VLDL-PL %		■					■
Age					■		
IDL-C %					■		
S-VLDL-TG					■		
L-HDL-FC %							■
L-VLDL-PL %							■
Omega-3		■					
Omega-6/Omega-3		■					
Gln		■					
Glycerol		■					
Creatinine		■					
L-HDL-PL %		■					
Acetate		■					
XXL-VLDL-PL		■					
M-HDL-PL		■					
M-LDL-CE %		■					

Red= features identified in >6 models

Bold= features identified in >3 models

LR: Logistic Regression

LR + Interactions (I)

RF: Random Forest

SVM: Support Vector Machine

NN: Neural Network

Table S5. Table showing all features identified by one or more models classifying RRMS vs SPMS. Related to Figure 1 and Table 2

Features	LR	LR + I	Bagged LR	Boosted LR	RF	SVM	NN	sPLS-DA	Direction SPMS
**Age**	■	■	■	■	■	■	■	■	↑
**EDSS**	■	■	■	■	■	■	■	■	↑
**Gln**	■	■	■	■	■	■	■	■	↑
**LA %**	■	■	■	■	■	■	■		↓
**M-LDL-CE %**		■	■	■	■	■	■		↑
**LA**	■	■	■	■	■	■			↑
**S-LDL-FC**	■	■	■	■	■	■		■	↑
**Acetoacetate**		■	■	■	■	■		■	↑
**SFA**		■	■	■	■	■		■	↑
**Omega-6 %**		■	■	■	■	■		■	↓
**MUFA %**		■	■	■	■	■		■	↑
**PUFA %**	■		■	■	■	■		■	↓
**XL-VLDL-FC %**			■	■	■	■	■	■	↓
**S-HDL-FC**			■	■	■	■		■	↑
**Lactate**	■	■					■	■	↑
**Val**	■	■					■	■	↓
**XXL-VLDL-FC %**	■	■					■	■	↓
**Tyr**	■	■					■	■	↓
**Citrate**	■	■					■	■	↑
**L-LDL-PL %**			■	■	■	■		■	↓
**Cholines**			■	■	■	■			↑
**Sphingomyelins**			■	■	■	■			↑
**IDL-TG**			■	■	■	■			↑
**S-HDL-PL**			■	■	■	■			↑
**Pyruvate**	■	■					■		↓
**XL-VLDL-C %**	■	■					■		↓
**M-LDL-PL %**	■	■					■		↓
**Glucose**		■					■	■	↓
**XS-VLDL-FC %**		■					■	■	↓
**S-LDL-TG**			■	■		■			↑
**IDL-PL %**	■	■							↓
**His**		■					■		↑
**L-VLDL-FC %**		■					■		↓
**XL-VLDL-TG %**		■			■				↑
**Ala**		■						■	↓
**XXL-VLDL-TG %**		■						■	↑
**L-VLDL-PL %**		■						■	↑
**L-VLDL-TG %**		■						■	↑
Smoking status	■						■		-
LDL size	■							■	↓
XXL-VLDL-PL %	■							■	↓
Gly								■	↓
ApoB/ApoA1		■							↑
SFA %		■							↑
Creatinine		■						■	↓
S-HDL-C %		■						■	↑
M-HDL-FC %		■							↑
Omega-3 %		■							↑
Omega-6/Omega-3		■							↓
IDL-CE %		■							↑
S-VLDL-CE %		■							↓
L-LDL-TG %	■								↓
Omega-3		■							↑
Ethnicity							■		-
bOHbutyrate								■	↑
Albumin								■	↑
S-VLDL-TG								■	↑
M-HDL-TG								■	↑
L-HDL-TG								■	↓
XS-VLDL-PL								■	↑
S-VLDL-CE								■	↑
XXL-VLDL-PL								■	↑
XS-VLDL-CE								■	↑
XL-HDL-TG								■	↑
XS-VLDL-PL %								■	↓
S-HDL-CE								■	↑
L-VLDL-C %								■	↓
S-VLDL-CE %								■	↓
M-VLDL-CE								■	↑
S-LDL-TG %								■	↑
S-HDL-TG								■	↑
M-VLDL-PL %								■	↓
IDL-P								■	↑

KEY:
LR: Logistic Regression
LR + Interactions (I): metabolites added to the table were from in. and ex. Age and EDSS models
RF: Random Forest
SVM: Support Vector Machine
NN: Neural Network
sPLS-DA: sparse Partial Least Squares Discriminant Analysis -metabolites added to the table were from in. and ex. Age and EDSS models
Red= features identified in >6 models
Bold= features identified in >3 models

Table S6: Top interactions and coefficients for logistic regression with interactions (LR+I) including and excluding age and EDSS for SPMS vs RRMS comparison. Related to Figure 1 and Figure S2.

LR+I including age and EDSS

Factor	Beta
Intercept	-1.1106599
ApoB/ApoA1:Age	0.0777074
LA:Pyruvate	-0.3398374
Omega-6 %:Val	-0.0584395
Omega-6 %:Glucose	-0.1933711
MUFA %:S-HDL-C %	0.05199317
SFA %:Gln	0.08796401
LA %:Tyr	-0.1127191
Gln:M-LDL-CE %	0.26057705
Gln:EDSS	0.08799802
His:Age	0.11331785
Val:XL-VLDL-C %	-0.0957238
Lactate:Age	0.54367062
Citrate:EDSS	0.18125666
Acetoacetate:EDSS	0.28674241
Creatinine:EDSS	0.41152852
S-LDL-FC:Age	0.41622108
XXL-VLDL-TG %:Age	0.21786332
XL-VLDL-TG %:Age	0.06416022
XL-VLDL-TG %:EDSS	0.18187136
XS-VLDL-FC %:M-LDL-PL %	-0.002853
IDL-PL %:M-LDL-PL %	-0.0285791
M-LDL-CE %:M-HDL-FC %	0.39117465
M-LDL-CE %:Age	0.04414799

LR+I excluding age and EDSS

Factor	Beta
Intercept	-1.084410151
ApoB/ApoA1:Acetoacetate	0.041561739
Omega-3:XXL-VLDL-TG %	0.175023892
SFA:His	0.006459898
LA:Pyruvate	-0.489451393
Omega-3 %:Acetoacetate	0.080075825
Omega-6 %:Glucose	-0.141861957
SFA %:Gln	0.258392678
LA %:Ala	-0.694986548
Omega-6/Omega-3:Pyruvate	-0.019649458
Gln:Lactate	0.727380474
Gln:XL-VLDL-TG %	0.300023722
His:Citrate	0.25537691
His:Acetoacetate	0.10444734
Val:XL-VLDL-C %	-0.052546335
Val:S-VLDL-CE %	-0.035689057
XXL-VLDL-FC %:L-VLDL-PL %	-0.211992623
L-VLDL-FC %:L-VLDL-TG %	0.146917332
XS-VLDL-FC %:M-LDL-PL %	-0.022137246
IDL-PL %:M-LDL-PL %	-0.115469559
IDL-CE %:M-LDL-CE %	0.310040011
M-LDL-CE %:M-HDL-FC %	0.55487113

Table S7: Top variables associated with stratification of patients with RRMS vs SPMS calculated using the Youden Index. Related to Figure 1D-E and Table S8

Feature	Cut-off	Unit	Sensitivity	Specificity	ROC	Youden J Statistic
Acetoacetate	>0.0172	mmol/l	0.966	0.788	0.873	0.754
SFA	>3.94	mmol/l	0.862	0.846	0.866	0.708
Glutamine	>0.635	mmol/l	0.931	0.731	0.861	0.662
S-HDL-FC	>0.122	mmol/l	0.897	0.731	0.859	0.628
Cholines	>2.74	mmol/l	0.793	0.827	0.836	0.620
S-HDL-PL	>0.658	mmol/l	0.862	0.731	0.800	0.593
Linoleic acid	>3.88	mmol/l	0.862	0.731	0.792	0.593
Sphinomyelins	>0.468	mmol/l	0.897	0.692	0.819	0.589
Omega-6	<38.8	%	0.759	0.827	0.853	0.586
IDL-TG	>0.092	mmol/l	0.793	0.750	0.802	0.543
XL-VLDL-FC	<11.4	%	0.931	0.596	0.783	0.527
MUFA	>24.9	%	0.655	0.865	0.807	0.520
PUFA	<43.6	%	0.793	0.712	0.829	0.505
M-LDL-CE	>48.0	%	0.828	0.673	0.785	0.501

The Youden index was calculated using the R package ‘OptimalCutpoints’ and the biomarker analysis tool in MetaboAnalyst 5.0.(Chong J et al*). See Methods

All features with a Youden Index >0.5 were included in the Autoscore analysis

*Chong J, Xia J. Using MetaboAnalyst 4.0 for Metabolomics Data Analysis, Interpretation, and Integration with Other Omics Data. In: Li S, editor. Computational Methods and Data Analysis for Metabolomics. New York, NY: Springer US; 2020. p. 337-60

Table S8: Autoscore training analysis. Related to Figure 1D-E and Table S7

Using the cut off values for the top five metabolites indicates that a combined score of >97 is associated with transition to SPMS

**Fine-tuned Scores: TRAIN SET*** (70% of patients)**			**TEST SET (30% of patients)**
==============	================	=====	AUC: 0.9464 95% CI: 0.8408-1 (DeLong)
variable	interval (expression value)	point	Score threshold: >= 97
==============	================	=====	Other performance indicators based on this score threshold:
Cholines	<2.7	0	Sensitivity: 1
	>=2.7	1	Specificity: 0.9286
			PPV: 0.9091
Gln	<0.563	0	NPV: 1
	[0.563,0.633)	24	
	>=0.633	27	
SFA	<3.21	0	
	[3.21,3.8)	22	
	>=3.8	24	
Acetoacetate	<0.00511	0	
	[0.00511,0.0169)	22	
	>=0.0169	25	
Sphingomyelins	<0.435	0	
	>=0.435	24	
==============	================	=====	

***Performance (based on validation set 70% patients, after fine-tuning): AUC: 0.9515   95% CI: 0.9003-1 (DeLong). Best score threshold: >= 97. Other performance indicators based on this score threshold: Sensitivity: 1

Table S9: Top 20 up and down regulated genes in patients with SPMS vs RRMS. Related to Figure 3

Gene ID	Annotation	P-value	log2FC
PFN1	Profilin 1	4.20E-83	3.308
DBNL	Drebrin like	1.12E-70	2.623
TBC1D10C	TBC1 domain family member 10C	3.16E-54	4.058
LIMD2	LIM domain containing 2	1.12E-40	2.658
HEXB	Hexosaminidase subunit beta	1.41E-40	2.727
MFSD1	Major facilitator superfamily domain containing 1	1.06E-38	2.149
CYTH2	Cytohesin 2	9.26E-38	2.335
EID1	EP300 interacting inhibitor of differentiation 1	4.37E-37	2.259
ELAC2	ElaC ribonuclease Z 2	9.28E-37	3.596
IKBKG	Inhibitor of nuclear factor kappa B kinase regulatory subunit gamma	6.50E-34	3.338
BAD	BCL2 associated agonist of cell death	7.82E-33	3.525
CFP	Complement factor properdin	5.22E-30	2.685
SPON2	Spondin 2	7.65E-30	4.163
TSC22D4	TSC22 domain family member 4	2.85E-29	2.638
MID1IP1	MID1 interacting protein 1	4.97E-28	2.881
MFSD10	Major facilitator superfamily domain containing 10	5.27E-28	3.056
TPST2	Tyrosylprotein sulfotransferase 2	7.75E-28	2.262
TRADD	TNFRSF1A associated via death domain	5.14E-27	2.706
CSK	C-terminal Src kinase	7.35E-27	2.388
NINJ1	Ninjurin 1	2.19E-26	2.06
ZNF384	Zinc finger protein 384	1.85E-13	-2.136
NRF1	Nuclear respiratory factor 1	2.01E-08	-1.15
CCR5	C-C motif chemokine receptor 5	4.66E-08	-1.241
ETS1	ETS proto-oncogene 1, transcription factor	8.02E-08	-1.763
CARD8-AS1	CARD8 antisense RNA 1	4.66E-07	-1.24
ARCN1	Archain 1	5.32E-07	-0.867
ABHD10	Abhydrolase domain containing 10, depalmitoylase	8.59E-07	-1.52
TNFSF8	TNF superfamily member 8	1.03E-06	-0.795
BMS1P20	BMS1 pseudogene 20	3.08E-06	-1.314
GOT2	Glutamic-oxaloacetic transaminase 2	1.47E-05	-0.617
A2M	Alpha-2-macroglobulin	1.85E-13	-2.136
MZB1	Marginal zone B and B1 cell specific protein	2.01E-08	-1.15
ZNF780A	Zinc finger protein 780A	4.66E-08	-1.241
ZNF124	Zinc finger protein 124	8.02E-08	-1.763
LINC00116 / MTLN	Mitoregulin	4.66E-07	-1.24
OSGEP	O-sialoglycoprotein endopeptidase	5.32E-07	-0.867
CSTB	Cystatin B	8.59E-07	-1.52
GLOD4	Glyoxalase domain containing 4	1.03E-06	-0.795
NPTN	Neuroplastin	3.08E-06	-1.314
LOC652276	Potassium channel tetramerization domain containing 5 pseudogene	1.47E-05	-0.617

Table S11: Genes (215) associated with the metabolic pathways (cellular respiration, aminoacyl-tRNA biosynthesis and glycerolipid metabolism) identified by MSEA. Related to Figure 2, 3 and Figure S4

ATP5F1D	QDPR	ST3GAL4	IMPDH1
ATP6V0C	CARM1	DDX1	QPRT
ATP6AP1	PRMT5	WDR4	PARP16
COX6C	SLC25A1	OSGEP	RFK
COX7B	HAGH	NUP107	TSEN54
CYC1	OGDH	NUP85	TP53RK
FXN	ACAA1	POLR2L	ELAC2
NDUFS3	NARS2	SCMH1	TRMT61A
PDK4	NAT10	HSPA13	TRMT6
UQCRB	CA4	CCR5	CTU2
UQCRFS1	CBR3	G3BP2	GRHPR
COX5A	CD1D	APOBR	YARS / YARS1
LRPPRC	NCAPH2	ACOT4	DBNL / ABP1
PHB2	TP53INP2	ABCD1	S100A8 / MIF
ISCU	RPL31	G0S2	PRMT6
HTRA2	BAD	HCCS	DLD / FAM3A
MRPL34	FUS	MTLN	HAGHL
MRPL11	NDUFB10	PDHX	BCKDHB
RHOT2	PFN1	MMP9	SLC16A3
TIMM50	RAB1A	KIF5B	PPIF
ANXA11	TNFRSF1A	SCD5	PGLS
PITPNM1	SQSTM1	PLA2G12A	AKR7A2
NCOR1	RB1CC1	BCKDK	IDH2
CERT1	NUP50	RPL34	AXIN1
AGPAT2	ATXN2L	RPL23	FADD
PNPLA6	ATG2A	GAMT	UBE2J2
STARD3	NUP210	PAPSS1	LHPP
SCAP	NXT1	ENOPH1	SLC2A1
SIN3B	UBQLN2	RPS3A	RTN3
PIP5K1C	UBQLN1	RPL7	CLTA
GDE1	WIPI1	RPL9	HDAC2
PIAS4	MAP1LC3B	RPS7	NRF1
INPP5K	MAP1LC3A	MAP2K7	BBC3
SMPD4	MGST1	PTPN1	ARNTL
PNPLA2	NFKBIA	RAF1	ARSA
MID1IP1	MAP2K2	RELA	CSNK1G2
MTMR14	ACBD6	IKBKG	GALC
CERS4	HELZ2	PRKD2	HEXB
PLBD1	MED30	NCF1	HSD17B1
RUFY1	OSBPL5	ATP6V1B2	INPP5D
CPTP	PTPMT1	GALK1	INPPL1
MED25	PLD4	NIPSNAP2	INSIG1
RPS27	CYP4F22	SNCA	ELOVL4
RPS27L	PIK3R6	ADPGK	VAPA
ACTN4	PHOSPHO1	DNAJC30	PSME3
SERPINE1	ABHD3	FOXK1	EEFSEC
CST3	SUMF1	SCO1	RPS24
SLC47A1	PLPP6	FDXR	
PPBP	PLCD1	POMC	
ADM	DGKZ	PPP1CC	
APRT	GPAA1	SRD5A1	
MRPL4	PIGQ	GLRX2	
UNG	NAAA	PRNP	
HGS	PLPPR2	HINT2	
TNFSF13B	PBX1	DUT	
GOT2	ST3GAL2	GUCY1B1	
